# Lung epithelium as a sentinel and effector system in pneumonia – molecular mechanisms of pathogen recognition and signal transduction

**DOI:** 10.1186/1465-9921-7-97

**Published:** 2006-07-08

**Authors:** Stefan Hippenstiel, Bastian Opitz, Bernd Schmeck, Norbert Suttorp

**Affiliations:** 1 Department of Internal Medicine/Infectious Diseases and Respiratory Medicine, Charité – Universitätsmedizin Berlin, 13353 Berlin, Germany

## Abstract

Pneumonia, a common disease caused by a great diversity of infectious agents is responsible for enormous morbidity and mortality worldwide. The bronchial and lung epithelium comprises a large surface between host and environment and is attacked as a primary target during lung infection. Besides acting as a mechanical barrier, recent evidence suggests that the lung epithelium functions as an important sentinel system against pathogens. Equipped with transmembranous and cytosolic pathogen-sensing pattern recognition receptors the epithelium detects invading pathogens. A complex signalling results in epithelial cell activation, which essentially participates in initiation and orchestration of the subsequent innate and adaptive immune response. In this review we summarize recent progress in research focussing on molecular mechanisms of pathogen detection, host cell signal transduction, and subsequent activation of lung epithelial cells by pathogens and their virulence factors and point to open questions. The analysis of lung epithelial function in the host response in pneumonia may pave the way to the development of innovative highly needed therapeutics in pneumonia in addition to antibiotics.

## Types of pneumonia, different types of pathogens, economic burden of pneumonia

Pneumonia is the third leading cause of death worldwide and the leading cause of death due to infectious disease in industrialized countries. In developing countries, approximately 2 million deaths (20% of all deaths) of children are due to pneumonia [1]. The majority of patients with community-acquired pneumonia (CAP) in industrialized countries are treated as outpatients with a low mortality rate usually less than 1%. In patients requiring inpatient management, the overall mortality rate increases up to approximately 12%. Of note, lethality rate in hospitalized patients differs significantly among different patient groups due to comorbidity (COPD, stroke, etc.) or risk factors (age, patients from nursing homes) [2].

In nosocomial pneumonia (hospital-acquired pneumonia, HAP; health-care associated pneumonia, HCAP) mortality increases substantially. HAP accounts for 15% of all nosocomial infections, its mortality rate exceeds 30%, although the attributable mortality is lower [3-5]. Requirement of mechanical ventilation is a high risk factor for the development of HAP with high mortality. This form of CAP, called ventilator-associated pneumonia (VAP) occurs in up to 47% of all intubated patients and varies among patient populations [6]. It definitely results in an increased length of stay. Moreover, high mortality rates are reported ranging from 34% in mixed medical/surgical intensive care unit patients [7] to up to 57.1% in heart surgical patients [8].

Consequently, CAP and HAP represent an enormous economic burden to the public health systems. CAP alone causes costs to the US economy of about US$ 20 billion in the United States [9] due to more than 10 million visits to physicians, 64 million days of restricted activity and over 600,00 hospitalizations per year [10].

Increasing antimicrobial resistance of pathogens causing CAP (e.g. *Streptococcus pneumoniae *[11,12]) and VAP (e.g. *Pseudomonas aerugenosa*, *Staphylococcus aureus *[6,13]) as well as the increasing number of humans with increased susceptibility to pneumonia (e.g. geriatric and/or immunocompromised people [14]) will aggravate the problem. Consequently, the development of new preventive and therapeutic strategies is urgently warranted.

Bacteria are the most common cause of pneumonia in adults. Most CAP-cases are due to infections with *S. pneumoniae*, *Haemophilus influenzae*, and *Mycoplasma pneumoniae *(Table 1) [15,16]. In patients with severe CAP, *Legionella *spp. as well as gram-negative bacilli and *S. aureus *have to be considered besides pneumococci [15,16]. The majority of late onset-VAP cases is caused by *S. aureus*, including antibiotic-resistant subtypes, *Pseudomonas *spp., *Klebsiella *spp., as well as *Acitenobacter *spp. [17].

**Table 1:** Important pathogens causing pneumonia

Pathogen	CAP	HAP/HCAP	Adults	Children
Bacteria				
*S. pneumoniae*	+++	+++	+++	+++
*H. influenzae*	++	++	++	++
*M. pneumoniae*	+++	+	++	+++
*Chlamydia *spp.	+		(+)	++
*Klebsiella *spp.	+	++		
*Legionella *spp.	++	+++		
*S. aureus*	++	+++	+++	+
*P. aerugenosa*	+	+++	+	
*Acinetobacter *spp.		++		
Viruses				
RSV	++		+	+++
Rhinovirus	++		(+)	++
Influenza virus	++	+	+	++
Parainfluenza virus	++		+	++
Fungi				
*Candida *spp.		++1		
*Aspergillus *spp.		++1		
*P. jirovecii*		+2		

+ indicates the relative importance of the pathogen and the frequency of isolation in adults or children. 1 of importance in immunocompromised hosts. 2 important opportunistic pathogen in HIV/AIDS patients. CAP, community-acquired pneumonia; HAP, hospital-acquired pneumonia; HCAP, health-care associated pneumonia. Based on collective data [2,5,6,15-18,23,252,253].

Interestingly, in children, a high rate of co-infections with viruses such as influenza A or B as well as respiratory syncytial virus (RSV) is observed in pneumococcal pneumonia [18]. Tsolia et al. recently provided evidence for high prevalence of viral infections, in particular rhinovirus infections, in school-age children hospitalized due to CAP [19]. Such infections have to be considered in the context of asthma attacks in children as well as in asthma and COPD exacerbations of adults [20-22].

Overall, in young infants, viruses such as RSV, parainfluenza and influenza virus are the most common cause of pneumonia (Table 1). In immunocompromised adults, in patients with asthma, chronic bronchitis or COPD, viruses are more frequently identified as the causative agent of pneumonia than in immunocompetent adult beings [23,24]. Cytomegalovirus-related pneumonia continues to be a major cause of morbidity and mortality in transplant recipients.

In addition to viruses, fungi like *Candida *spp. or *Aspergillus *spp. induce pneumonia in the immunocompromised host (post-transplantation, post-chemotherapy, etc.) [25]. Pneumonia due to infections with the opportunistic pathogen *Pneumocystis jirovecii *(former *P. carinii*) is a major cause of illness and death in HIV/AIDS patients [26].

The new millennium added previously unrecognized respiratory viral pathogens to the list of pneumonia-causing agents [27]. Human metapneumovirus might be the causative agent in up to 12% of young children suffering from severe respiratory tract illness [28,29]. Avian influenza A viruses, especially subtype H5N1, originally seen in Southeast Asia, has caused more than one hundred cases of severe pneumonia due to direct bird-to-human transmissions [30,31]. Moreover, human coronaviruses causing severe acute respiratory syndrome (SARS) as well as two other isolates (HcoV-NL and HcoV-HKU1) were identified in the last years [30,32,33]. Thus, a number of important emerging and reemerging pathogens have to be added to the list of pneumonia causing agents.

## The pulmonary innate immune system

A large variety of pathogens are known to cause pneumonia. The innate immune system serves as the first line host defense system against invading pathogens. Localized at the interface between the environment and the host, the airway epithelium does not only form a large mechanical barrier, but it is also predisposed as a sentinel system to detect pathogens entering via the airways and to initiate the initial host immunological response.

Pseudostratified and columnar tracheobronchial epithelium consisting of ciliated cells, secretory goblet cells and cells with microvilli provide mechanisms for mucocilliary clearance. In the bronchioles, cuboidal epithelium and secretory clara cells line the airways. Alveolar type I cells and type II cells constitute the alveolar epithelium. About 95% of the internal lung surface is built by alveolar type I cells. Fused to endothelial cells by their basement membranes both cell types together form the gas exchange barrier. Alveolar type II cells fulfil many known functions, including the regulation of the lung surfactant system [34], alveolar fluid content [35], and are important for the replacement of injured type I cells [34,36]. Although not evaluated systematically, it seems predictable that differentiated lung epithelial cells from different origin in the lung will have a cell-type specific response to a given pathogen. This might be due to varying expression of pattern recognition receptors (PRR), and/or cell-specific protein expression (e.g. surfactant protein expression) [37] as well as to different susceptibility to injury [38].

Although all pathogens causing pneumonia may directly interact with tracheobronchial as well as alveolar epithelium, the molecular mechanisms and consequences of these interactions are poorly understood. For some of the important pathogens mentioned, little or nothing is known about the consequences of epithelial infection.

Taking the enormous global burden of pneumonia, the increasing number of antibiotic- resistant bacteria, and the emergence of new pulmonary pathogens into account, an exact analysis of molecular mechanisms of disease is mandatory to form a rational basis for the development of innovative interventional procedures in pneumonia. In this review we focus on current molecular aspects of pathogen-lung epithelial interactions.

## Recognition of entering pathogens by lung epithelium

A prerequisite for the initiation of host responses is the recognition of pathogens by the host immune system. A tremendous progress in this field was the discovery that the 10 germline-encoded human TLRs comprising the TLR family act as transmembraneous pattern recognition receptors (PRR) detecting a large variety of conserved pathogen-associated molecular pattern (PAMP) as well as presumably even self-molecules [39-43]. TLR activation initiates expression of important mediators of the subsequent immune response. In addition, recent research points to the existence of cytosolic PRRs, which may serve as a second sentinel system detecting particularly but not exclusively invasive pathogens. These include members of the NACHT (domain present in NAIP, CIITA, HET-E, TP-1)-LRR (leucine-rich repeats) (NLR) family [44-46], as well as the caspase-recruitment domain (CARD)-containing RNA-helicases retinoic acid inducible gene-I (RIG-I) and melanoma differentiation-associated gene 5 (MDA5) [47,48]. Both, the TLRs and the NLRs, but not the CARD-helicases, possess LRR domains, which seem to be crucial for pathogen recognition.

## Transmembraneous receptors

**Figure 1:**
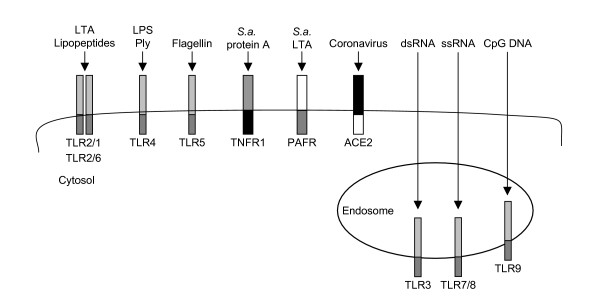
Transmembraneous receptors involved in lung epithelial cell recognition of pathogens. Heterodimers composed of TLR2/TLR1 or TLR2/TLR6 recognize lipoproteins and lipoteichoic acid. TLR4 detects LPS and bacterial factors like pneumococcal pneumolysin (Ply). Flagellin, an integral structure of bacterial flagella, is recognized by TLR5. Although not acting as classical PRRs in principle, TNF receptor-1 (TNFR1) and platelet activating factor receptor (PAFR) displayed an important role in *S. aureus *induced pneumonia by recognition of staphylococci protein A or LTA, respectively. In addition, SARS causing coronavirus is detected by angiotensin converting enzyme 2 (ACE2) in the lung epithelium. Transmembraneous TLRs residing within the endosome of some cells detect dsRNA (TLR3), ssRNA (TLR7/8) or CpG DNA (TLR9).

In brief, TLR1, TLR2, and TLR6 are at least partly located on the cell surface, and may collaborate to discriminate between the molecular structures of triacyl and diacyl lipopeptides, as well as lipoteichoic acid [49-52]. TLR4 recognizes bacterial lipopolysaccharide (LPS) [53], whereas TLR5 detects bacterial flagellin on the cell surface [54]. In contrast, TLR3 [55], TLR7, TLR8 [56,57] and TLR9 [58] are located in endosomal compartments and perceive microbial nucleic acids: TLR3 recognizes viral dsRNA, whereas TLR7 and TLR8 recognize viral single stranded (ss)RNA. Bacterial and viral cytosine-phosphate-guanosine (CpG)-containing DNA motives are recognized by TLR9. The ligand for TLR10 has not been identified yet [59,60] (Fig. 1).

Distribution and subcellular expression of TLRs differ between immune cells and epithelial cells. Most results, however, were obtained by analysis of different (immortalized) cell lines and a systematic exploration of TLR receptor expression in healthy human lungs or inflamed human lungs is still missing.

In cultured human lung epithelial cells, mRNA of all 10 TLRs has been detected [61,62]. Moreover, TLR1-5 as well as TLR9 protein was shown to be expressed in tracheal and bronchial epithelial cell lines [61]. Expression of TLR2, TLR4, and TLR5 has been documented *in vivo *in human airway epithelial cells [63-65] as well as TLR2 expression in alveolar epithelial cells [66].

Besides lung epithelial cells hematopoietic cells (resident in the lung or infiltrating during the host-pathogen combat) also contribute to the host response in pneumonia. Studies analyzing global responses in pneumonia by using TLR-deficient mice (or C3H/Hej mice, which express a non-functional TLR4), therefore give only limited information on the role of lung epithelial TLR expression in pneumonia. Furthermore, most studies published focused on e.g. lethality, global bacterial burden or immune cell recruitment. Nevertheless, studies by Wang et al. [67] and Chu et al. [68] demonstrated important epithelium-related information obtained from these models by specific analysis of the lung epithelium. Thus, Wang et al. showed that *H. influenza *induced TLR4-dependent TNFα and MIP1α expression in lung airway epithelial cells *in vivo *[67]. Moreover, by the use of TLR2-deficient mice Chu et al. reported reduced airway mucin expression in *M. pneumoniae *infected TLR2-deficient mice [68].

The expression and localization of TLRs may differ between lung epithelial and classical immune cells. For example, TLR4 apparently is not expressed on the surface of the tracheobronchial epithelial cell line BEAS-2B and the alveolar epithelial cell line A549. In these cells – which only responded to purified TLR4 ligand LPS in much higher doses than e.g. macrophages-TLR4 seemed to be expressed in a intracellular compartment [69] although contradictory results were published as well [61]. It was suggested that under inflammatory conditions a re-localisation of TLR4 to the cell membrane with subsequent increasing susceptibility to LPS took place as documented by studies using RSV infected lung epithelial cells [70]. Nevertheless, an increasing number of studies clearly indicate that lung epithelial cells are sufficiently activated by a broad variety of TLR ligands [39,40,71].

Lipoteichoic acid [72], commercially available peptidoplycan [73], and *M. pneumoniae *[68] activated cultured human pulmonary epithelial cells in a TLR2-dependent manner. Results obtained with *S. pneumoniae*-infected epithelial cells indicated a cooperative recognition of these bacteria by TLR1 and TLR2 but not by TLR2 and TLR6 [74]. *P. aeruginosa *flagella as well as the C-terminus of its cytotoxin ExoS stimulated lung epithelial cells TLR2 and TLR5-dependently [75]. In an elegant study, Soong et al. showed that lipid rafts-associated complexes of TLR2 and asialoGM1 presented at the surface of airway epithelial cells formed broadly responsive signalling complexes reactive to important lung pathogens like *P. aerugenosa *or *S. aureus *[76]. Notably, by using TLR2-deficient mice, the role of TLR2 for *M. pneumoniae*-induced airway mucin expression was demonstrated recently [68]. Taken together, TLR2 represents an important functionally active PRR on the surface of lung epithelial cells.

Double-stranded RNA, a byproduct of viral replication, is recognized by TLR3 within the endocytoplasmic compartment. Thus, TLR3 reportedly participates in the recognition of influenza A virus [77], rhinovirus [78] and detects the synthetic viral dsRNA analog polyribocytidylic acid [poly(I:C)] [78,79] in lung epithelial cells. Moreover, in a model of RSV infection in TLR3-deficient mice, Rudd et al. demonstrated that TLR3 was not required for viral clearance in the lung, but it had a large impact on mucus production [80].

TLR4 contributes to the recognition of various bacterial pathogens by lung epithelial cells [61,69,72]. In *H. influenza *infection, activation of the transcription factor NF-κB and subsequent TNFα and MIP1α expression was reduced in lung epithelial cells of TLR4-deficient mice compared to wild-type cells, demonstrating the critical role of TLR4 *in vivo *for epithelial cell activation by this pathogen [67]. Consistent with this notion, two common, co-segregating missense mutations (Asp299Gly and Thr399Ile) affecting the extracellular domain of TLR4 reduced the response to inhaled LPS in humans [81]. Besides LPS, other pathogen-derived factors may also be recognized by lung epithelial TLR4. For example, the important pneumococcal virulence factor pneumolysin was found to induce a TLR4-dependent activation of epithelial cells [74,82] and chlamydial heat shock protein also initiated TLR4- and TLR2-related signalling [83,84]. In addition, TLR4 together with CD14 might be involved in the recognition of RSV fusion protein, thereby contributing to anti-viral host defence in the lung [85]. Accordingly, TLR4 mutations (Asp299Gly and Thr399IIe) may be associated with increased risk of severe RSV bronchiolitis in human infants, thus implicating a role of TLR4 in this virus infection [86].

Flagellin is a major structural component of flagella, a locomotive organell present on a wide range of bacteria [87]. It induces TLR5-dependent signalling on the surface of host cells, which might also involve TLR4 [87]. Lung epithelial cells were stimulated by flagella of e.g. *Bordetella bronchiseptica *[88], *P. aerugenosa *[65,89], and *L. pneumophila *[90]. The importance of this interaction was highlighted by the observation that a common dominant TLR5 stop codon polymorphism leading to impaired flagellin signalling is associated with increased susceptibility to Legionaires' disease [90].

In contrast to TLR2-6, little is known about the expression and function of TLR7-8 in lung epithelium. However, TLR6 may function in heterodimers with TLR2 thereby contributing to the recognition of diacylated lipoproteins [41-43]. It is not clear if lung epithelium expresses functionally active TLR7 and TLR8 although these receptors recognize guanosine- and uridine-rich single-stranded (ss)RNA found in many viruses.

Functionally active TLR9 was expressed in the human alveolar tumour epithelial cell line A549 as demonstrated by Droemann et al [66]. Although immunization of mice with CpG motives reduced the burden of *Cryptococcus neoformans *in the lung, it is unclear if this effect was dependent on lung epithelial TLR9, or more likely, induced by TLR9-expressing immune cells causing promotion of a sufficient Th1-type immune response [91]. However, promotion of lung TLR9 signalling by using synthetic agonists may enhance the host defence and may even be beneficial in patients with acquired immune deficiency.

From an analytical perspective the use of purified virulence factors has been essential for understanding PRR function. However, infection of lung epithelial cells with "complete" pathogens containing different PAMPs results in a more complex, but also more realistic stimulation (e.g. pneumococci possesses TLR2-stimulating LTA [92] as well as TLR4-stimulating pneumolysin [74,82]). In addition, more than one TLR may be activated by one PAMP as demonstrated for the bifunctional type-III secreted cytotoxin ExoS from *P. aerogenosa*, which was shown to activate both, TLR2 and TLR4 signalling [93].

The situation is furthermore complicated by the fact that pathogens may modulate the expression pattern of TLRs and induce a re-localization of the PRRs. For example, pneumococci increased the expression of TLR1 and TLR2 in bronchial epithelial cells, but displayed no effect on TLR4 and TLR6 expression [74]. In mice, inhalation of LPS induced a strong increase in TLR4 protein expression in the bronchial epithelium as well as in macrophages within 24 hours [94]. Poly(I:C) may elevate the expression of TLR1-3 but decrease the expression of TLR5 and TLR6 [79]. Increased expression as well as membrane localization of TLR3 [95] and TLR4 [70] have been observed after RSV infection of airway epithelial cells. The effect of mixed infections with different pathogens (e.g. influenza virus and pneumococci) on TLR expression/localization and subsequent cell activation is widely unknown (see below). Thus, during an infection process, the recognition of pathogens is a dynamic process influenced by varying TLR expression on pulmonary epithelium. Furthermore, the liberation of cytokines (e.g. TNF-α, IFNγ) during the initiated host response as well as therapeutic interventions (e.g. corticosteroids) influences expression of TLRs [96].

Of note, besides the traditional membranous PRRs, other membraneous receptor molecules may also be critically involved in epithelial activation by pathogens (Fig. 1). *S. aureus *protein A binds to TNFR1 presented on airway epithelial cells thereby inducing pneumonia [97]. In addition, stimulation of platelet-activating factor receptor by *S. aureus *LTA, and subsequent epidermal growth factor receptor activation may stimulate mucus expression and cell activation in lung epithelium independently of TLR2 and TLR4 [98]. Angiotensin converting enzyme 2 (ACE2) expressed in the lung has recently been identified as a potential SARS coronavirus receptor and SARS and the Spike protein of this virus reduced the expression of ACE2 [99,100]. Notably, blocking of the renin-angiotensin pathway reduced the worsening of disease induced by injection of Spike protein in mice [100]. Thus, non-classical pathogen-recognizing transmembranous receptors may also be important for the pathophysiology of pneumonia.

## Cytosolic receptors

**Figure 2:**
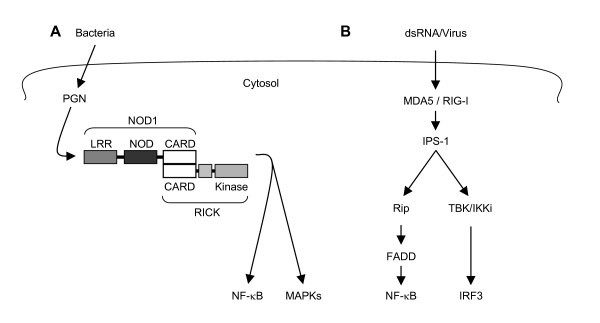
Recognition of pathogens by cytosolic PRRs. (A) As an example, NOD1 is shown. NOD1 is activated by peptidoglycan-derived peptides. The carboxy-terminal LRR domain is involved in agonist recognition, whereas the central NOD (NACHT) domain has ATPase activity and facilitates self-oligomerization. At the amino-terminal a protein-protein interaction mediating caspase-recruitment domain (CARD) is localized (one CARD domain in NOD1, two in NOD2). Recruitment of the kinase-activity containing adaptor molecule RICK transmits the signal to the NF-κB pathway and it may also participate in MAPK stimulation. (B) The cytosolic PRRs MDA5 and RIG-I recognize dsRNA leading to a complex signalling pathway involving molecules like IPS-1, Rip, FADD promoting NF-κB activation, whereas IPS, TBK and IKKi mediate IRF3 activation.

Various bacterial lung pathogens like *C. pneumoniae *[101,102], *L. pneumophila *[103,104], and *S. pneumonia *[105,106] are able to invade and replicate efficiently within epithelial cells. Inside the cells, these pathogens are protected against detection and attack by various defense mechanisms of the innate immune system. Not only whole bacteria are sensed intracellularly, the same is true for bacterial proteins or genetic material after injection into host cells via various bacterial secretion systems (e.g. type III or IV secretion system) [107-110]. Moreover, many viruses replicate very efficiently within the lung epithelium. Recent research provided evidence that cytosolic PRRs exist which detect these invasive pathogens and initiate an appropriate immune response [44-46] (Fig. 2).

The human NLR family, currently consisting of 22 proteins, contains NALP (NACHT-, LRR-, and pyrin domain-containing proteins), NOD (nucleotide-binding oligomerization domain), CIITA (class II transactivator), IPAF (ICE-protease activating factor) and NAIP (neuronal apoptosis inhibitor protein). These proteins are implicated in the detection of intracellular pathogens or other general danger signals [44-46]. Two of the best characterized members of the NLRs are NOD1 and NOD2 [44,45,111]. In general, the importance of NOD proteins has been highlighted by the findings that critical mutations are associated with inflammatory granulomatous disorders (e.g. Chrohn's disease, Blau syndrome) [112]. In addition, an insertion-deletion polymorphism of the NOD1 gene effecting the LRR domain has been associated with asthma and high IgE levels as suggested recently [113,114].

NOD proteins share a tripartite domain structure: The carboxy-terminal LRR domain seems to mediate ligand recognition (Fig. 2). The central NOD (NACHT) domain exhibits ATPase activity and facilitates self-oligomerization. An amino-terminal localized caspase-recruitment domain (CARD) (one CARD domain in NOD1, two in NOD2) mediates protein-protein interaction [44-46].

NOD1 is activated by peptidoglycan-derived peptides containing γ-D-glutamyl-*meso*-diaminopimelic acid found mainly in Gram-negative bacteria [115,116], whereas NOD2 mediates responsiveness to the muramydipeptide MurNAc-L-Ala-D-isoGln conserved in peptidoglycans of basically all bacteria [117,118]. However, as for many of the TLRs and their agonists, there is no formal proof for the binding of the peptidoglycan motifs to the LRR domains of NOD1 and NOD2.

So far it is unclear how cytoplasmic NODs find their ligands: Some bacteria such as *Shigella *and *Listeria *reach the free cytosol of host cells [119]. Furthermore, injection of peptidoglycan-derived molecules in the host cell cytosol by type IVb secretion system-expressing bacteria (e.g. *L. pneumophila *[109]) has also to be considered since this mechanism was evidenced in experiments with *Helicobacter pylori *[110]. In addition, the peptide transporter PEPT1 was suggested to play a role in the uptake of muramyldipeptide and subsequent proinflammatory intestinal epithelial cell activation [120]. Thus, it is reasonable to speculate that the high-affinity peptide transporter PEPT2 expressed in the respiratory tract epithelium [121] is involved in NOD-peptidoglycan-related lung cell activation.

Although residing in the cytosol, it was shown that in intestinal epithelium, membrane recruitment of NOD2 was essential for NF-κB activation by muramyl dipeptide [122]. As known so far, NOD1 is ubiquitously expressed whereas NOD2 is primarily found in antigen presenting cells and epithelial cells. In human lung epithelium, we detected expression of NOD1 and lower expression of NOD2 in resting human BEAS-2B cells [106]. Further analysis revealed that intracellular pneumococci were recognized by NOD2 but not by NOD1 in epithelial cells. Moreover, NOD1 was implicated in lung infections with *P. aerugenosa *[123], and NOD2 in *Mycobacterium tuberculosis *infection [124]. In addition, our unpublished experiments indicated an important role of NOD1 in lung epithelial cell activation by *L. pneumophila*. Moreover, the respiratory pathogen *C. pneumoniae *activated human endothelial cells via NOD1 suggesting a role of this molecule also in lung infection [125]. The observation that NOD1 was involved in infection with *H. pylori *[110] and *Listeria monocytogenes *[126] further strengthened the hypothesis that NOD proteins act as important cytosolic PRRs.

After infection of pulmonary epithelial cells with *S. pneumoniae*, expression of NOD1 and NOD2 increased in these cells *in vitro *and overall expression was up-regulated in mouse lungs infected with pneumococci [106]. IFNγ, has been shown to increase NOD1 expression in epithelial cells [127], and TNFα as well as IFNγ, up-regulated expression of NOD2 [128]. Thus, as already explained for TLRs, the expression of cytosolic PRRs may also vary during the hassle with pathogens and the subsequent activation of the host immune system.

Besides NOD1 and NOD2, additional members of the NLR family may have a role in pneumonia. For example, *L. pneumophila *replicates in macrophages derived from A/J mice, but not in cells derived form other mouse-inbred strains. The higher susceptibility of A/J mice towards *Legionella *infection has been attributed to sequence differences and reduced expression of the NLR protein Naip5 (Birc1e) [129,130]. Accordingly, recent studies demonstrated that Naip5 together with IPAF or ASC recognizes *Legionella *flagellin and controls intracellular replication of *Legionella *within mice macrophages, and mediates IL-1β secretion, respectively [131-133]. Thus, at least in mice, bacterial flagellin is recognized by both, TLR5 on the cell surface and Naip5 within the cytosol.

As a great number of other members of the NLR protein family, such as NALP proteins (with exception of NALP10) also contain LRR domains implicated in pathogen recognition, additional members of this family may function as cytosolic PRRs or may be involved in inflammatory signalling [44,45,134,135]. For example, Nalp3/cryoporin has recently been demonstrated to mediate IL-1β and IL-18 secretion induced by a diverse variety of stimuli such as bacterial or viral RNA, muramyl dipeptide, TLR agonists, together with ATP, native bacteria (e.g. *S. aureus*) and bacterial toxins [136-139].

An important question is how activation of transmembranous and cytosolic receptors acts together in host cell responses. For example, a synergistic stimulation of cytokine induction by NOD1 or NOD2, together with TLRs has been observed in human dendritic and monocytic cells [140-143], while NLR proteins may act as inhibitors of TLR signalling. Overexpression of the NALP12 for example was shown to reduce TLR2/4- and *M. tuberculosis*-related activation of myeloid/monocytic cells [144]. Moreover, *in vivo *studies in NOD2-deficient mice or mice carrying a common Crohn's disease-associated NOD2 mutation yielded controversial results regarding functional NOD2/TLR2 interaction [145-147].

dsRNA is produced as an intermediate product during virus replication and recent observations point to the existence of cytosolic PRRs recognizing viral dsRNA (Fig. 2). Both, RIG-I and MDA5 recognizes dsRNA leading to activation of an antiviral response [47,48]. RIG-I and MDA5 comprise a carboxy-terminal DexD/H-box RNA helicase domain which seems to mediate recognition of dsRNA, whereas amino-terminal CARD domains mediate the recruitment of downstream signalling adaptor molecules [47,48]. Matikainen et al. reported that IFNβ and TNFα induced the expression of RIG-I in A549 cells. Expression of dominant-negative form of RIG-I inhibited influenza A virus-related activation of an IFNβ promoter suggesting a role of lung epithelial RIG-I in host defense [148]. Very recent studies in mice deficient in RIG-I or MDA5 indicated that RIG-I mediated IFN response to RNA viruses including influenza virus and MDA5 recognized picornavirus-infection [149]. Increased susceptibility of RIG-I-deficient mice towards influenza virus infection highlights the importance of this molecule for lung infection [149].

Besides these studies, however, nothing more is currently known about the expression of these molecules and their functional role in lung epithelial inflammation and disease.

## Downstream signalling pathways

The recognition of PAMPs by PRRs activates a network of signal transduction pathways. Although it is reasonable to suggest that most of these pathways function in pulmonary epithelial cells and in classical immune cells similarly in principle, most data have not been verified in human lung epithelial cells or in the lung *in vivo*. In the following, a brief introduction in basic mechanisms is given with special emphasis on signalling pathways known to be operative in lung epithelium.

In general, a central aspect of inflammatory activation by PRRs is the stimulation of NF-κB-dependent gene transcription [40,44,59,60]. On the other hand, increasing evidence points to an important role of interferon-regulating factor (IRF)-dependent gene transcription leading to the generation of type I interferons (IFN) and subsequent expression of co-called IFN-stimulated genes (ISGs) [150-152].

**Figure 3:**
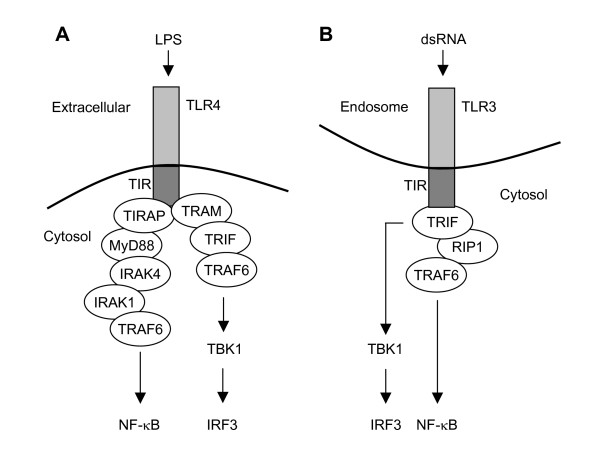
TLRs mediate activation of NF-κB- and IRF-related gene transcription. (A) Examples of recruited adaptor molecules critical for TLR4 function. With the possible exception of TLR3, all TLRs share a MyD88-dependent pathway for the activation of NF-κB. A protein complex composed of TIRAP, MyD88, IRAK4, IRAK1 and TRAF6 mediates NF-κB stimulation. In addition, TRAM, TRIF as well as TRAF6 and TBK1 stimulate IRF3 activation. (B) Located in the endosomal membrane, TLR3 recognizes dsRNA. Whereas TRIF recruitment connects TLR3 via TBK1 to IRF3 activation, further recruitment of RIP1 and TRAF6 stimulates NF-κB.

The ability of the TLRs to activate transcription factors leading to gene transcription differs and depends on differential engagement of the four TIR (Toll-interleukin-1 receptor) domain containing adaptor molecules MyD88 (differentiation primary response gene 88), TIRAP (toll-IL-1R domain-containing adaptor protein; Mal), TRIF (Toll/IL-1R domain-containing adaptor inducing IFNβ) and TRAM (Fig. 3). Thus, whereas all TLRs except TLR3 engage MyD88 in order to activate NF-κB and AP-1 [153,154], only TLR3 and TLR4 signal via TRIF and TRIF/TRAM, respectively, leading to additional activation of IRF3 and potentially IRF7 [155-158]. The forth adaptor TIRAP is recruited to TLR2 as well as TLR4 and is involved in the MyD88-dependent transcriptional activation of NF-κB [159,160]. In case of the conserved MyD88-dependent signalling leading to NF-κB activation, further signalling molecules, such as IRAK4 (interleukin-1 receptor-associated kinase-4), IRAK1, as well as TRAF6 (tumor necrosis factor receptor-associated factor-6), are additionally recruited downstream of MyD88 to the receptor complex [43,59]. Downstream of TLR7-9, a similar signalling module leads to the activation of IRF5 and IRF7 [161-165].

Small GTP binding Rho proteins like Rac1 may also participate in TLR-driven NF-κB dependent gene transcription, as recently shown for pneumococci infected human lung epithelial cells [74]. The canonical NF-κB pathway downstream the TLRs involves phosphorylation of IκB molecules sequestering NF-κB in the cytosol in unstimulated cells by the IKK (IκB kinase) complex finally leading to the proteosomal-mediated degradation of IκB [59,166]. Free NF-κB molecules translocate into the nucleus and initiate NF-κB dependent gene transcription [59,166].

Stimulation of this NF-κB activation was observed e.g. after infection of lung epithelial cells with pneumococci [74,167], *Moraxella catharrhalis *[168], *P. carinii *[169], *P. aerogenosa *[170], or exposure to purified virulence factors like LPS [171]. In addition to stimulation of transmembraneous TLRs, activation of NOD1 and NOD2 also results in NF-κB activation. Both NODs recruit the adaptor molecule RICK/Rip2 through CARD-CARD interaction [172,173] and we recently implicated the downstream signalling molecules IRAK1, IRAK2, TRAF6 as well as NIK (NF-κB-inducing kinase), TAB2 (transforming growth factor-β activated kinase binding protein) and TAK1 (transforming growth factor-β activated kinase) in *S. pneumoniae *initiated NOD2-dependent NF-κB activation in epithelial cells [106].

The important role of NF-κB activation for lung inflammation was furthermore emphasised by Sadikot et al., who demonstrated that selective overexpression of constitutively active IκB kinase in airway epithelial cells by adenoviral vectors was sufficient to induce NF-κB activation, inflammatory mediator production and neutrophilic lung inflammation in mice [174]. Moreover, by using the same experimental approaches, this group showed most recently that inflammatory signalling through NF-κB in lung epithelium is critical for proper innate immune response to *P. aeruginosa *[175]. In addition, inhibition of NF-κB by airway epithelium selective overexpression of an IκB suppressor reduced the inflammatory response upon intranasal application of LPS [171]. Overall, NF-κB activation is a central event in pathogen exposed lung epithelium.

As mentioned above, a key feature of some but not all TLRs is the initiation of IRF-dependent gene transcription. The cytosolic PRRs RIG-I and MDA5 are also capable to induce IRF3 and IRF7 activation [47,48] (Fig. 2). However, in contrast to the well-established canonical NF-κB pathway, the mechanisms of IRF activation are much more elusive and require further investigation. The complexity of these pathways may be illustrated by exemplarily focussing on IRF3, which is crucial for e.g. initial IFNβ expression. Different molecules like IFNβ promoter stimulator 1 (IPS-1, also known as MAVS, VISA, Cardif) (Fig. 2), TBK1, IKKi, or PI3 kinase pathway are implicated in the IRF3 activation process [176-182]. Activation of IRFs is vital for the regulation of type I (IFNα-subtyps, IFNβ, -ε, -κ, -ω) expression, participating in the host response against viruses and, notably, intracellular bacteria [183,184]. Besides acting on classical immune cells, expression of type I IFNs resulted in auto- and paracine stimulation of cells through specific receptors (IFNAR), stimulation of janus kinases, STATs, and subsequent expression of ISGs in epithelial cells [183,184]. Thus, although intracellular bacteria and viruses are important lung pathogens, neither the expression of central signalling molecules nor the resulting signalling events are known to date in lung epithelial cells.

Another important signalling pathway involves mitogen-activated protein kinases (MAPK). Pro-inflammatory signalling induced by several TLRs [59,185] as well as NOD1 and NOD2 involves the activation of ERK (extracellular signal-regulated kinase), JNK (c-Jun N-terminal kinase), and p38 MAPK [126,145,186]. Activation of these kinases was also observed e.g. in pneumococci- [74,167] or virus-infected [187] lung epithelium and in pneumococci-infected mice lungs [167].

**Figure 4:**
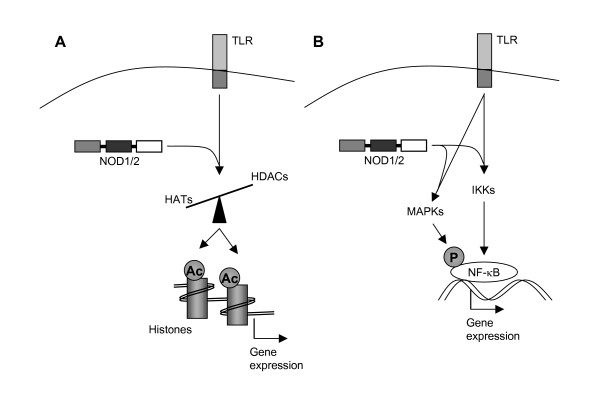
Histone modifications regulate the accessibility of the DNA to transcription factors. (A) In most cases, hyperacetylation (Ac) of histones loosens DNA-histone interaction thereby making gene promoters amenable for the binding of transcription factors. After stimulation of transmembraneous (e.g. TLRs) or cytosolic (e.g. NODs) PRRs histone acetylases (HATs) may be recruited whereas histone deacetylases (HDACs) may disappear resulting in increased histone acetylation. (B) In addition, after binding of the transcription factors to the DNA further modification of the bound transcription factor by PRR-mediated MAPK-dependent phosphorylation may be necessary to induce recruitment of the basal transcription apparatus of the cell and subsequent gene transcription as shown for pneumococci infected pulmonary epithelial cells.

The finding that e.g. the p38 MAPK pathway converges with the NF-κB pathway in IL-8 regulation illustrates the complex signalling network in infected lung epithelial cells: Blockade of p38 MAPK activity did not affect pneumococci-induced nuclear translocation and recruitment of NF-κB/RelA to the *il8 *promoter but reduced the level of phosphorylated RelA (serine 536) at the *il8 *promoter [167]. The inhibition of serine 536-RelA phosphorylation blocked pneumococci-mediated recruitment of RNA polymerase II (Pol II) to *il8 *promoter thereby averting IL-8 expression [167] (Fig. 4). Thus, p38 MAP kinase contributes to pneumococci-induced chemokine transcription by modulating p65 NF-κB-mediated transactivation in human lung epithelial cells.

DNA in euchromatin must be processed to allow for access of activated transcription factors. Increasing evidence indicates that histone modifications may serve as combinatorial code for the transcriptional activity state of genes in many cellular processes by loosening the DNA-histone interaction and unmasking of transcription factor binding sites [188]. In chromatin, 146 base pairs of DNA are wrapped in 1.65 turns around a histone octamer (H2A, H2B, H3, H4)_2 _[189]. A wide range of specific covalent modifications of accessible N-terminal histone tails are decisive for transcription repression or gene activation [190]. To date, acetylation (mostly lysine), phosphorylation (serine/threonine), methylation (lysine), ADP-ribosylation, and ubiquitination of histones have been described [191,192]. Phosphorylation at Ser-10 on H3 and acetylation at Lys-14 of H4 seem to have a special impact on gene regulation [189]. For example, it was found that LPS stimulation of dendritic cells induced p38 MAPK-dependent phosphorylation at Ser-10 on H3 and acetylation at Lys-14 on H4 specifically occurs at *il8*, and *mcp1*, but not at *tnfα *or *mip1α *genes [193]. Both modifications have been correlated with the immediate early gene induction. In addition, *L. monocytogenes*-related recruitment of histone acetylase (HAT), CBP and Pol II to the *il8 *promotor and subsequent *il8 *gene expression in human endothelial cells depended on p38 MAPK-related acetylation (Lys-8) of histone H4 and phosphorylation/acetylation (Ser-10/Lys-14) of histone H3 at the *il8 *promoter [194]. Furthermore, we recently demonstrated that *M. catharrhalis *enhanced global acetylation of histone H3 and H4 and at the *il8 *gene in human bronchial epithelial cells [168]. For this infection, global histone deacetylase (HDAC) expression as well as its activity decreased [168]. Considering that patients with chronic obstructive pulmonary disease (COPD) which are often colonized by *Moraxella *also display decreased HDAC activity [195,196], acute and chronic effects of histone-related (epigenetic) modifications should be taken into account in lung infection.

Besides the signaling pathways mentioned, other pathways, including e.g. tyrosine kinases [197] or protein kinase C [198], may also play an important role, but have not been analyzed yet in detail in pulmonary epithelium.

Importantly, most investigations focused on the effects purified virulence factors (e.g. LPS) or – at the most – of one pathogen. This approach does not take into account that mixed or sequential infections with different pathogens (e.g. influenza virus and pneumococci) causing severe pneumonia may occur. In a sequential infection model RSV infection lead to impaired clearance of *S. pneumoniae*, *S. aureus *or *P. aerugenosa *[199]. In addition, reduced clearance of pneumococci was observed after influenza A virus infection [200]. Polymicrobial colonization of lung epithelial cells by pneumococci and *H. influenzae *led to strong NF-κB activation and synergistic IL-8 expression and synergistic inflammation in mice *in vivo *[202]. Virus infection in concert with endogenous pro-inflammatory mediators may alter PRR expression in lung epithelium as evidenced for TLR3 [201] and RIG-I [148]. Thus, co-infections or mixed infections certainly will influence pathogen recognition, signal transduction and host gene transcription thereby opening up an important new field of research.

In conclusion, a complex network of signalling events is started through the recognition of pathogens by lung epithelial cells.

## Consequences for lung epithelial cell activation

The complex response of the lung epithelium to pathogen recognition reflects the great variety of stimuli and signalling pathways activated. The epithelial response includes production and secretion of inflammatory mediators such as cytokines and chemokines, the up-regulation of epithelial cell surface adhesion molecules as well as the enhanced liberation of antimicrobial peptides [39,40,71,203,204].

For example, a broad variety of purified virulence factors (e.g. flagella [75], LPS [72], LTA [72]) as well as complete bacteria (e.g. *S. pneumoniae *[74,106,167], *P. aerugenosa *[62,76], *S. aureus *[62], *M. catharrhalis *[168]) induced the liberation of the chemotactic cytokine IL-8, which is considered to play an important role in lung inflammation [205]. Agonists of e.g. TLR2, TLR4 and TLR9 stimulated the expression of TNFα as well as IL-6 by lung epithelium [61,70,96].

In addition, the pathogen-related liberation of cytokines by epithelial cells results in auto- and paracrine stimulation of further inflammation-regulating mediators. Systematic analysis of TNFα and IL-1β exposed primary human bronchial epithelial cells by cDNA representational difference analysis discovered over 60 regulated genes including proteases and antiproteases, adhesion molecules, as well as cyto- and chemokines [206].

Up-regulation of adhesion molecules like intercellular adhesion molecule 1 (ICAM-1) or vascular cell adhesion molecule-1 (VCAM-1) in pulmonary epithelium was observed after exposure to diverse stimuli such as LPS [40,61,207], outer membrane protein A from *K. pneumoniae *[208] or infection with *P. carinii *[209]. The liberation of immunodulatory cyto- and chemokines and up-regulation of adhesion molecules mediates the acute immune response by e.g. recruitment of leucocytes to the site of infection and modulates the initiation of adaptive immune response. In addition, systemic effects of lung epithelial inflammation by the release of e.g. granulocyte-macrophage colony-stimulating factor (GM-CSF) by activation of immature precursor cells have to be considered [210]. GM-CSF secretion was shown in *S. pneumoniae*-infected bronchial epithelial cells as well as in pneumococci-infected mice lungs [167].

Antimicrobial substances like defensins and cathelicidins secreted by pulmonary epithelium [203] are capable of killing Gram-positive and -negative bacteria, some fungi as well as enveloped viruses [211-213]. Some of these factors, like human β-defensin (hBD)-2 have shown to be up-regulated by cytokines as well as by bacteria like *P. aerogenosa *in lung epithelial cells [214].

In addition, inflamed epithelium may show increased arachidonic acid metabolism. In pneumococci-infected lung epithelium as well as in pneumococci-infected mice lung increased cyclooxygenase-2 expression and subsequently increased prostaglandin E_2 _(PGE_2_) liberation was noted [215]. PGE_2 _in turn may influence immune cells, blood perfusion distribution as well as lung function [216].

The epithelium thereby closely interacts with other cellular components of the innate immune system such as phagocytes (neutrophils, macrophages), natural killer cells and others [217-221]. Of note, today the exact contribution of parenchymal lung versus hematopoietic cells to the initiation and control of the immune response within the lung is not entirely clear and seems to be pathogen-specific as evidenced by studies using chimeric mouse models. In *P. aerugenosa*-infected mice lungs, expression of MyD88 in non-bone marrow derived cells is required for the early control of infection, including cytokine production and neutrophil recruitment, whereas on the long run both, parenchymal and hematopoietic cells were required to control pathogen replication [222]. After inhalation of endotoxin, the cytokine response seems to be mediated by hematopoietic cells in a myeloid differentiation primary response gene (88) (MyD88)-dependent way, whereas bronchoconstriction depended on resident cells as indicated by experiments with chimeric mice [223]. In studies using TLR4-deficient chimeric mice, expression of TLR4 on hematopoietic cells and macrophages seemed crucial to initiate the LPS-induced recruitment of neutrophils within the alveolar space [224]. On the other hand, inhibition of the nuclear factor-κB (NF-κB) pathway in distal lung epithelium lead to reduced neutrophilic lung inflammation and cytokine expression [171,225].

In addition, the interaction of lung epithelium with hematopoietic cells may alter the immune response of both cell types. Transmigration across lung epithelial cells decreased apoptosis of polymorphonuclear leucocytes [226,227] and migration over the surface of alveolar epithelial cells facilitated alveolar macrophage phagocytic activity in a ICAM-1-dependent manner [228]. On the other hand, LPS-exposed mononuclear phagocytes induced the expression of human β-defensin-2 in lung epithelial cells, thereby strengthening the epithelial innate immune response [229]. Moreover, the complex interaction is further highlighted by the observation that defensins produced by neutrophils may stimulate the release of cytokines by epithelial cells and promote epithelial cell proliferation [230-232].

Overall, it is reasonable to suggest that the pulmonary epithelium contributes significantly to the initiation of an appropriate immune response in pneumonia. Furthermore, the transition of the innate to adaptive immune response might significantly be modulated by epithelial-related actions. Finally, although poorly examined and not discussed here, the lung epithelium may also possess mechanisms to negatively control and terminate inflammatory responses [233].

## Concluding remarks

Pulmonary epithelium is well equipped to act as an interactive sentinel system detecting entering pathogens. Recognition of pathogens or their products by transmembraneous and intracellular receptors activated signalling cascades leading to a complex activation status of pulmonary epithelium and influences local and systemic immune response. Although pneumonia is a common worldwide disease, causing millions of deaths annually, central mechanisms of pathogen-lung epithelial interaction are still obscure. Basic questions, like the expression of functional active transmembraneous and cytosolic PRRs in normal and inflamed human lungs, are widely unanswered. Results about PRR function are often obtained in classical immune cells and transferred to pulmonary lung epithelial cells function although important differences may exist (e.g. TLR4 expression and localization). For many important lung pathogens only fragmentary information about their interaction is available. In addition, the modulatory role of alveolar fluid containing immunregulatory surfactant proteins needs further investigation [234-237]. Finally, the complexity, which is introduced by co-infections and subsequent infection must be appreciated in further studies.

Overall, it seems imperative to accelerate the verification of important general mechanisms of innate immunity for the organ lung with respect to pneumonia. In addition, the lung is a unique organ and it is important to identify organ specific mechanisms of innate immunity. The relative ease of transnasal or tracheal application of small interference RNA might allow a relatively fast verification of important newly identified molecules *in vivo *without the time-consuming establishment of knock out models [238-240].

In addition to the analysis of host response initiation, the understanding of control mechanisms of local inflammation within the lung (resolution of inflammation, repair mechanisms) is crucial [241-243]. In the lung, a high degree of organ function must be preserved on a minute basis to allow for sufficient gas exchange. In this sense, lungs differ from gut or kidney, because inflammation in the lungs must be controlled much more tightly. Of note, as noticed for the intestinal epithelium [244,245], lung epithelial PAMP recognition may be somewhat restricted to avoid frequent epithelial-mediated inflammation. Ambient air contains bacteria and endotoxin [246], and the aerosolized concentrations of e.g. endotoxin is increased in e.g. agricultural environments [247,248]. Limitation of pro-inflammatory lung epithelial activation may be due to restriction of PRR expression on epithelial surfaces [69,70], reduced expression of co-signalling molecules (as shown for e.g. MD-2 [249]), or increased expression of inhibitory molecules (e.g. TOLLIP [250,251]).

Overall, there are a lot of important questions about the molecular mechanisms by which the lung epithelium acts in pneumonia. Their analysis may help to develop future innovative therapeutic strategies in pneumonia.

## References

[B1] Williams BG, Gouws E, Boschi-Pinto C, Bryce J, Dye C (2002). Estimates of world-wide distribution of child deaths from acute respiratory infections. Lancet Infect Dis.

[B2] Mandell LA (2004). Epidemiology and etiology of community-acquired pneumonia. Infect Dis Clin North Am.

[B3] Chastre J, Trouillet JL, Vuagnat A, Joly-Guillou ML, Clavier H, Dombret MC, Gibert C (1998). Nosocomial pneumonia in patients with acute respiratory distress syndrome. Am J Respir Crit Care Med.

[B4] Fagon JY, Chastre J, Hance AJ, Montravers P, Novara A, Gibert C (1993). Nosocomial pneumonia in ventilated patients: a cohort study evaluating attributable mortality and hospital stay. Am J Med.

[B5] Lynch JPIII (2001). Hospital-acquired pneumonia: risk factors, microbiology, and treatment. Chest.

[B6] Shaw MJ (2005). Ventilator-associated pneumonia. Curr Opin Pulm Med.

[B7] Fowler RA, Lapinsky SE, Hallett D, Detsky AS, Sibbald WJ, Slutsky AS, Stewart TE (2003). Critically ill patients with severe acute respiratory syndrome. JAMA.

[B8] Bouza E, Perez A, Munoz P, Jesus PM, Rincon C, Sanchez C, Martin-Rabadan P, Riesgo M (2003). Ventilator-associated pneumonia after heart surgery: a prospective analysis and the value of surveillance. Crit Care Med.

[B9] Marrie TJ (1994). Community-acquired pneumonia. Clin Infect Dis.

[B10] Dixon RE (1985). Economic costs of respiratory tract infections in the United States. Am J Med.

[B11] Bishai WR (2005). Clinical significance of pneumococcal resistance and factors influencing outcomes. Treat Respir Med.

[B12] Ferrara AM (2005). New fluoroquinolones in lower respiratory tract infections and emerging patterns of pneumococcal resistance. Infection.

[B13] Combes A, Luyt CE, Fagon JY, Wollf M, Trouillet JL, Gibert C, Chastre J (2004). Impact of methicillin resistance on outcome of Staphylococcus aureus ventilator-associated pneumonia. Am J Respir Crit Care Med.

[B14] Janssens JP (2005). Pneumonia in the elderly (geriatric) population. Curr Opin Pulm Med.

[B15] File TM (2003). Community-acquired pneumonia. Lancet.

[B16] Gant V, Parton S (2000). Community-acquired pneumonia. Curr Opin Pulm Med.

[B17] Kollef MH (2005). Bench-to-bedside review: antimicrobial utilization strategies aimed at preventing the emergence of bacterial resistance in the intensive care unit. Crit Care.

[B18] Michelow IC, Olsen K, Lozano J, Rollins NK, Duffy LB, Ziegler T, Kauppila J, Leinonen M, McCracken GHJ (2004). Epidemiology and clinical characteristics of community-acquired pneumonia in hospitalized children. Pediatrics.

[B19] Tsolia MN, Psarras S, Bossios A, Audi H, Paldanius M, Gourgiotis D, Kallergi K, Kafetzis DA, Constantopoulos A, Papadopoulos NG (2004). Etiology of community-acquired pneumonia in hospitalized school-age children: evidence for high prevalence of viral infections. Clin Infect Dis.

[B20] Hayden FG (2004). Rhinovirus and the lower respiratory tract. Rev Med Virol.

[B21] Johnston SL (2005). Overview of virus-induced airway disease. Proc Am Thorac Soc.

[B22] Tan WC (2005). Viruses in asthma exacerbations. Curr Opin Pulm Med.

[B23] Greenberg SB (2002). Respiratory viral infections in adults. Curr Opin Pulm Med.

[B24] Ison MG, Fishman JA (2005). Cytomegalovirus pneumonia in transplant recipients. Clin Chest Med.

[B25] Pound MW, Drew RH, Perfect JR (2002). Recent advances in the epidemiology, prevention, diagnosis, and treatment of fungal pneumonia. Curr Opin Infect Dis.

[B26] Thomas CFJ, Limper AH (2004). Pneumocystis pneumonia. N Engl J Med.

[B27] Fouchier RA, Rimmelzwaan GF, Kuiken T, Osterhaus AD (2005). Newer respiratory virus infections: human metapneumovirus, avian influenza virus, and human coronaviruses. Curr Opin Infect Dis.

[B28] Crowe JEJ (2004). Human metapneumovirus as a major cause of human respiratory tract disease. Pediatr Infect Dis J.

[B29] Williams JV, Harris PA, Tollefson SJ, Halburnt-Rush LL, Pingsterhaus JM, Edwards KM, Wright PF, Crowe JEJ (2004). Human metapneumovirus and lower respiratory tract disease in otherwise healthy infants and children. N Engl J Med.

[B30] Chen H, Deng G, Li Z, Tian G, Li Y, Jiao P, Zhang L, Liu Z, Webster RG, Yu K (2004). The evolution of H5N1 influenza viruses in ducks in southern China. Proc Natl Acad Sci U S A.

[B31] Li KS, Guan Y, Wang J, Smith GJ, Xu KM, Duan L, Rahardjo AP, Puthavathana P, Buranathai C, Nguyen TD, Estoepangestie AT, Chaisingh A, Auewarakul P, Long HT, Hanh NT, Webby RJ, Poon LL, Chen H, Shortridge KF, Yuen KY, Webster RG, Peiris JS (2004). Genesis of a highly pathogenic and potentially pandemic H5N1 influenza virus in eastern Asia. Nature.

[B32] Fouchier RA, Hartwig NG, Bestebroer TM, Niemeyer B, de Jong JC, Simon JH, Osterhaus AD (2004). A previously undescribed coronavirus associated with respiratory disease in humans. Proc Natl Acad Sci U S A.

[B33] Wang JT, Chang SC (2004). Severe acute respiratory syndrome. Curr Opin Infect Dis.

[B34] Fehrenbach H (2001). Alveolar epithelial type II cell: defender of the alveolus revisited. Respir Res.

[B35] Matthay MA, Folkesson HG, Clerici C (2002). Lung epithelial fluid transport and the resolution of pulmonary edema. Physiol Rev.

[B36] Uhal BD (1997). Cell cycle kinetics in the alveolar epithelium. Am J Physiol.

[B37] Crouch E, Wright JR (2001). Surfactant proteins a and d and pulmonary host defense. Annu Rev Physiol.

[B38] Nakamura M, Matute-Bello G, Liles WC, Hayashi S, Kajikawa O, Lin SM, Frevert CW, Martin TR (2004). Differential response of human lung epithelial cells to fas-induced apoptosis. Am J Pathol.

[B39] Basu S, Fenton MJ (2004). Toll-like receptors: function and roles in lung disease. Am J Physiol Lung Cell Mol Physiol.

[B40] Greene CM, McElvaney NG (2005). Toll-like receptor expression and function in airway epithelial cells. Arch Immunol Ther Exp (Warsz ).

[B41] Janeway CAJ, Medzhitov R (2002). Innate immune recognition. Annu Rev Immunol.

[B42] Kawai T, Akira S (2005). Pathogen recognition with Toll-like receptors. Curr Opin Immunol.

[B43] Takeda K, Kaisho T, Akira S (2003). Toll-like receptors. Annu Rev Immunol.

[B44] McDonald C, Nunez G, Inohara, Chamaillard (2005). NOD-LRR proteins: role in host-microbial interactions and inflammatory disease. Annu Rev Biochem.

[B45] Martinon F, Tschopp J (2005). NLRs join TLRs as innate sensors of pathogens. Trends Immunol.

[B46] Philpott DJ, Girardin SE (2004). The role of Toll-like receptors and Nod proteins in bacterial infection. Mol Immunol.

[B47] Andrejeva J, Childs KS, Young DF, Carlos TS, Stock N, Goodbourn S, Randall RE (2004). The V proteins of paramyxoviruses bind the IFN-inducible RNA helicase, mda-5, and inhibit its activation of the IFN-beta promoter. Proc Natl Acad Sci U S A.

[B48] Yoneyama M, Kikuchi M, Natsukawa T, Shinobu N, Imaizumi T, Miyagishi M, Taira K, Akira S, Fujita T (2004). The RNA helicase RIG-I has an essential function in double-stranded RNA-induced innate antiviral responses. Nat Immunol.

[B49] Aliprantis AO, Yang RB, Mark MR, Suggett S, Devaux B, Radolf JD, Klimpel GR, Godowski P, Zychlinsky A (1999). Cell activation and apoptosis by bacterial lipoproteins through toll-like receptor-2. Science.

[B50] Buwitt-Beckmann U, Heine H, Wiesmuller KH, Jung G, Brock R, Akira S, Ulmer AJ (2006). TLR1- and TLR6-independent recognition of bacterial lipoeptides. J Biol Chem.

[B51] Opitz B, Schroder NW, Spreitzer I, Michelsen KS, Kirschning CJ, Hallatschek W, Zahringer U, Hartung T, Gobel UB, Schumann RR (2001). Toll-like receptor-2 mediates Treponema glycolipid and lipoteichoic acid-induced NF-kappaB translocation. J Biol Chem.

[B52] Takeuchi O, Kawai T, Muhlradt PF, Morr M, Radolf JD, Zychlinsky A, Takeda K, Akira S (2001). Discrimination of bacterial lipoproteins by Toll-like receptor 6. Int Immunol.

[B53] Poltorak A, He X, Smirnova I, Liu MY, Van Huffel C, Du X, Birdwell D, Alejos E, Silva M, Galanos C, Freudenberg M, Ricciardi-Castagnoli P, Layton B, Beutler B (1998). Defective LPS signaling in C3H/HeJ and C57BL/10ScCr mice: mutations in Tlr4 gene. Science.

[B54] Hayashi F, Smith KD, Ozinsky A, Hawn TR, Yi EC, Goodlett DR, Eng JK, Akira S, Underhill DM, Aderem A (2001). The innate immune response to bacterial flagellin is mediated by Toll-like receptor 5. Nature.

[B55] Alexopoulou L, Holt AC, Medzhitov R, Flavell RA (2001). Recognition of double-stranded RNA and activation of NF-kappaB by Toll-like receptor 3. Nature.

[B56] Diebold SS, Kaisho T, Hemmi H, Akira S, Sousa R (2004). Innate antiviral responses by means of TLR7-mediated recognition of single-stranded RNA. Science.

[B57] Heil F, Hemmi H, Hochrein H, Ampenberger F, Kirschning C, Akira S, Lipford G, Wagner H, Bauer S (2004). Species-specific recognition of single-stranded RNA via toll-like receptor 7 and 8. Science.

[B58] Hemmi H, Takeuchi O, Kawai T, Kaisho T, Sato S, Sanjo H, Matsumoto M, Hoshino K, Wagner H, Takeda K, Akira S (2000). A Toll-like receptor recognizes bacterial DNA. Nature.

[B59] Akira S, Takeda K (2004). Toll-like receptor signalling. Nat Rev Immunol.

[B60] Beutler B (2005). The Toll-like receptors: analysis by forward genetic methods. Immunogenetics.

[B61] Greene CM, Carroll TP, Smith SG, Taggart CC, Devaney J, Griffin S, O'neill SJ, McElvaney NG (2005). TLR-induced inflammation in cystic fibrosis and non-cystic fibrosis airway epithelial cells. J Immunol.

[B62] Muir A, Soong G, Sokol S, Reddy B, Gomez MI, Van Heeckeren A, Prince A (2004). Toll-like receptors in normal and cystic fibrosis airway epithelial cells. Am J Respir Cell Mol Biol.

[B63] Hauber HP, Tulic MK, Tsicopoulos A, Wallaert B, Olivenstein R, Daigneault P, Hamid Q (2005). Toll-like receptors 4 and 2 expression in the bronchial mucosa of patients with cystic fibrosis. Can Respir J.

[B64] Hertz CJ, Wu Q, Porter EM, Zhang YJ, Weismuller KH, Godowski PJ, Ganz T, Randell SH, Modlin RL (2003). Activation of Toll-like receptor 2 on human tracheobronchial epithelial cells induces the antimicrobial peptide human beta defensin-2. J Immunol.

[B65] Zhang Z, Louboutin JP, Weiner DJ, Goldberg JB, Wilson JM (2005). Human airway epithelial cells sense Pseudomonas aeruginosa infection via recognition of flagellin by Toll-like receptor 5. Infect Immun.

[B66] Droemann D, Albrecht D, Gerdes J, Ulmer AJ, Branscheid D, Vollmer E, Dalhoff K, Zabel P, Goldmann T (2005). Human lung cancer cells express functionally active Toll-like receptor 9. Respir Res.

[B67] Wang X, Moser C, Louboutin JP, Lysenko ES, Weiner DJ, Weiser JN, Wilson JM (2002). Toll-like receptor 4 mediates innate immune responses to Haemophilus influenzae infection in mouse lung. J Immunol.

[B68] Chu HW, Jeyaseelan S, Rino JG, Voelker DR, Wexler RB, Campbell K, Harbeck RJ, Martin RJ (2005). TLR2 signaling is critical for Mycoplasma pneumoniae-induced airway mucin expression. J Immunol.

[B69] Guillot L, Medjane S, Le Barillec K, Balloy V, Danel C, Chignard M, Si-Tahar M (2004). Response of human pulmonary epithelial cells to lipopolysaccharide involves Toll-like receptor 4 (TLR4)-dependent signaling pathways: evidence for an intracellular compartmentalization of TLR4. J Biol Chem.

[B70] Monick MM, Yarovinsky TO, Powers LS, Butler NS, Carter AB, Gudmundsson G, Hunninghake GW (2003). Respiratory syncytial virus up-regulates TLR4 and sensitizes airway epithelial cells to endotoxin. J Biol Chem.

[B71] Chaudhuri N, Dower SK, Whyte MK, Sabroe I (2005). Toll-like receptors and chronic lung disease. Clin Sci (Lond).

[B72] Armstrong L, Medford AR, Uppington KM, Robertson J, Witherden IR, Tetley TD, Millar AB (2004). Expression of functional toll-like receptor-2 and -4 on alveolar epithelial cells. Am J Respir Cell Mol Biol.

[B73] Gon Y, Asai Y, Hashimoto S, Mizumura K, Jibiki I, Machino T, Ra C, Horie T (2004). A20 inhibits toll-like receptor 2- and 4-mediated interleukin-8 synthesis in airway epithelial cells. Am J Respir Cell Mol Biol.

[B74] Schmeck B, Huber S, Moog K, Zahlten J, Hocke AC, Opitz B, Hammerschmidt S, Mitchell TJ, Kracht M, Rosseau S, Suttorp N, Hippenstiel S (2005). Pneumococci induced TLR- and Rac1-dependent NF-{kappa}B-recruitment to the IL-8 promoter in lung epithelial cells. Am J Physiol Lung Cell Mol Physiol.

[B75] Adamo R, Sokol S, Soong G, Gomez MI, Prince A (2004). Pseudomonas aeruginosa flagella activate airway epithelial cells through asialoGM1 and toll-like receptor 2 as well as toll-like receptor 5. Am J Respir Cell Mol Biol.

[B76] Soong G, Reddy B, Sokol S, Adamo R, Prince A (2004). TLR2 is mobilized into an apical lipid raft receptor complex to signal infection in airway epithelial cells. J Clin Invest.

[B77] Guillot L, Le Goffic R, Bloch S, Escriou N, Akira S, Chignard M, Si-Tahar M (2005). Involvement of toll-like receptor 3 in the immune response of lung epithelial cells to double-stranded RNA and influenza A virus. J Biol Chem.

[B78] Hewson CA, Jardine A, Edwards MR, Laza-Stanca V, Johnston SL (2005). Toll-like receptor 3 is induced by and mediates antiviral activity against rhinovirus infection of human bronchial epithelial cells. J Virol.

[B79] Ritter M, Mennerich D, Weith A, Seither P (2005). Characterization of Toll-like receptors in primary lung epithelial cells: strong impact of the TLR3 ligand poly(I:C) on the regulation of Toll-like receptors, adaptor proteins and inflammatory response. J Inflamm (Lond).

[B80] Rudd BD, Smit JJ, Flavell RA, Alexopoulou L, Schaller MA, Gruber A, Berlin AA, Lukacs NW (2006). Deletion of TLR3 alters the pulmonary immune environment and mucus production during respiratory syncytial virus infection. J Immunol.

[B81] Arbour NC, Lorenz E, Schutte BC, Zabner J, Kline JN, Jones M, Frees K, Watt JL, Schwartz DA (2000). TLR4 mutations are associated with endotoxin hyporesponsiveness in humans. Nat Genet.

[B82] Malley R, Henneke P, Morse SC, Cieslewicz MJ, Lipsitch M, Thompson CM, Kurt-Jones E, Paton JC, Wessels MR, Golenbock DT (2003). Recognition of pneumolysin by Toll-like receptor 4 confers resistance to pneumococcal infection. Proc Natl Acad Sci U S A.

[B83] Costa CP, Kirschning CJ, Busch D, Durr S, Jennen L, Heinzmann U, Prebeck S, Wagner H, Miethke T (2002). Role of chlamydial heat shock protein 60 in the stimulation of innate immune cells by Chlamydia pneumoniae. Eur J Immunol.

[B84] Da Costa CU, Wantia N, Kirschning CJ, Busch DH, Rodriguez N, Wagner H, Miethke T (2004). Heat shock protein 60 from Chlamydia pneumoniae elicits an unusual set of inflammatory responses via Toll-like receptor 2 and 4 in vivo. Eur J Immunol.

[B85] Kurt-Jones EA, Popova L, Kwinn L, Haynes LM, Jones LP, Tripp RA, Walsh EE, Freeman MW, Golenbock DT, Anderson LJ, Finberg RW Pattern recognition receptors TLR4 and CD14 mediate response to respiratory syncytial virus.

[B86] Tal G, Mandelberg A, Dalal I, Cesar K, Somekh E, Tal A, Oron A, Itskovich S, Ballin A, Houri S, Beigelman A, Lider O, Rechavi G, Amariglio N (2004). Association between common Toll-like receptor 4 mutations and severe respiratory syncytial virus disease. J Infect Dis.

[B87] Honko AN, Mizel SB (2005). Effects of flagellin on innate and adaptive immunity. Immunol Res.

[B88] Lopez-Boado YS, Cobb LM, Deora R (2005). Bordetella bronchiseptica flagellin is a proinflammatory determinant for airway epithelial cells. Infect Immun.

[B89] Sadikot RT, Blackwell TS, Christman JW, Prince AS (2005). Pathogen-host interactions in Pseudomonas aeruginosa pneumonia. Am J Respir Crit Care Med.

[B90] Hawn TR, Verbon A, Lettinga KD, Zhao LP, Li SS, Laws RJ, Skerrett SJ, Beutler B, Schroeder L, Nachman A, Ozinsky A, Smith KD, Aderem A (2003). A common dominant TLR5 stop codon polymorphism abolishes flagellin signaling and is associated with susceptibility to legionnaires' disease. J Exp Med.

[B91] Edwards L, Williams AE, Krieg AM, Rae AJ, Snelgrove RJ, Hussell T (2005). Stimulation via Toll-like receptor 9 reduces Cryptococcus neoformans-induced pulmonary inflammation in an IL-12-dependent manner. Eur J Immunol.

[B92] Schroder NW, Morath S, Alexander C, Hamann L, Hartung T, Zahringer U, Gobel UB, Weber JR, Schumann RR (2003). Lipoteichoic acid (LTA) of Streptococcus pneumoniae and Staphylococcus aureus activates immune cells via Toll-like receptor (TLR)-2, lipopolysaccharide-binding protein (LBP), and CD14, whereas TLR-4 and MD-2 are not involved. J Biol Chem.

[B93] Epelman S, Stack D, Bell C, Wong E, Neely GG, Krutzik S, Miyake K, Kubes P, Zbytnuik LD, Ma LL, Xie X, Woods DE, Mody CH (2004). Different domains of Pseudomonas aeruginosa exoenzyme S activate distinct TLRs. J Immunol.

[B94] Saito T, Yamamoto T, Kazawa T, Gejyo H, Naito M (2005). Expression of toll-like receptor 2 and 4 in lipopolysaccharide-induced lung injury in mouse. Cell Tissue Res.

[B95] Rudd BD, Burstein E, Duckett CS, Li X, Lukacs NW (2005). Differential role for TLR3 in respiratory syncytial virus-induced chemokine expression. J Virol.

[B96] Homma T, Kato A, Hashimoto N, Batchelor J, Yoshikawa M, Imai S, Wakiguchi H, Saito H, Matsumoto K (2004). Corticosteroid and cytokines synergistically enhance toll-like receptor 2 expression in respiratory epithelial cells. Am J Respir Cell Mol Biol.

[B97] Gomez MI, Lee A, Reddy B, Muir A, Soong G, Pitt A, Cheung A, Prince A (2004). Staphylococcus aureus protein A induces airway epithelial inflammatory responses by activating TNFR1. Nat Med.

[B98] Lemjabbar H, Basbaum C (2002). Platelet-activating factor receptor and ADAM10 mediate responses to Staphylococcus aureus in epithelial cells. Nat Med.

[B99] Imai Y, Kuba K, Rao S, Huan Y, Guo F, Guan B, Yang P, Sarao R, Wada T, Leong-Poi H, Crackower MA, Fukamizu A, Hui CC, Hein L, Uhlig S, Slutsky AS, Jiang C, Penninger JM (2005). Angiotensin-converting enzyme 2 protects from severe acute lung failure. Nature.

[B100] Kuba K, Imai Y, Rao S, Gao H, Guo F, Guan B, Huan Y, Yang P, Zhang Y, Deng W, Bao L, Zhang B, Liu G, Wang Z, Chappell M, Liu Y, Zheng D, Leibbrandt A, Wada T, Slutsky AS, Liu D, Qin C, Jiang C, Penninger JM (2005). A crucial role of angiotensin converting enzyme 2 (ACE2) in SARS coronavirus-induced lung injury. Nat Med.

[B101] Krull M, Maass M, Suttorp N, Rupp J (2005). Chlamydophila pneumoniae. Mechanisms of target cell infection and activation. Thromb Haemost.

[B102] Jahn HU, Krull M, Wuppermann FN, Klucken AC, Rosseau S, Seybold J, Hegemann JH, Jantos CA, Suttorp N (2000). Infection and activation of airway epithelial cells by Chlamydia pneumoniae. J Infect Dis.

[B103] Cianciotto NP, Stamos JK, Kamp DW (1995). Infectivity of Legionella pneumophila mip mutant for alveolar epithelial cells. Curr Microbiol.

[B104] Mody CH, Paine RIII, Shahrabadi MS, Simon RH, Pearlman E, Eisenstein BI, Toews GB (1993). Legionella pneumophila replicates within rat alveolar epithelial cells. J Infect Dis.

[B105] Cundell DR, Gerard NP, Gerard C, Idanpaan-Heikkila I, Tuomanen EI (1995). Streptococcus pneumoniae anchor to activated human cells by the receptor for platelet-activating factor. Nature.

[B106] Opitz B, Puschel A, Schmeck B, Hocke AC, Rosseau S, Hammerschmidt S, Schumann RR, Suttorp N, Hippenstiel S (2004). Nucleotide-binding oligomerization domain proteins are innate immune receptors for internalized Streptococcus pneumoniae. J Biol Chem.

[B107] Mota LJ, Sorg I, Cornelis GR (2005). Type III secretion: the bacteria-eukaryotic cell express. FEMS Microbiol Lett.

[B108] Pizarro-Cerda J, Cossart P (2006). Bacterial adhesion and entry into host cells. Cell.

[B109] Segal G, Feldman M, Zusman T (2005). The Icm/Dot type-IV secretion systems of Legionella pneumophila and Coxiella burnetii. FEMS Microbiol Rev.

[B110] Viala J, Chaput C, Boneca IG, Cardona A, Girardin SE, Moran AP, Athman R, Memet S, Huerre MR, Coyle AJ, DiStefano PS, Sansonetti PJ, Labigne A, Bertin J, Philpott DJ, Ferrero RL (2004). Nod1 responds to peptidoglycan delivered by the Helicobacter pylori cag pathogenicity island. Nat Immunol.

[B111] Inohara N, Nunez G (2003). NODs: intracellular proteins involved in inflammation and apoptosis. Nat Rev Immunol.

[B112] Kambe N, Nishikomori R, Kanazawa N (2005). The cytosolic pattern-recognition receptor Nod2 and inflammatory granulomatous disorders. J Dermatol Sci.

[B113] Hysi P, Kabesch M, Moffatt MF, Schedel M, Carr D, Zhang Y, Boardman B, von Mutius E, Weiland SK, Leupold W, Fritzsch C, Klopp N, Musk AW, James A, Nunez G, Inohara N, Cookson WO (2005). NOD1 variation, immunoglobulin E and asthma. Hum Mol Genet.

[B114] Weidinger S, Klopp N, Rummler L, Wagenpfeil S, Novak N, Baurecht HJ, Groer W, Darsow U, Heinrich J, Gauger A, Schafer T, Jakob T, Behrendt H, Wichmann HE, Ring J, Illig T (2005). Association of NOD1 polymorphisms with atopic eczema and related phenotypes. J Allergy Clin Immunol.

[B115] Chamaillard M, Hashimoto M, Horie Y, Masumoto J, Qiu S, Saab L, Ogura Y, Kawasaki A, Fukase K, Kusumoto S, Valvano MA, Foster SJ, Mak TW, Nunez G, Inohara N (2003). An essential role for NOD1 in host recognition of bacterial peptidoglycan containing diaminopimelic acid. Nat Immunol.

[B116] Girardin SE, Boneca IG, Carneiro LA, Antignac A, Jehanno M, Viala J, Tedin K, Taha MK, Labigne A, Zahringer U, Coyle AJ, DiStefano PS, Bertin J, Sansonetti PJ, Philpott DJ (2003). Nod1 detects a unique muropeptide from gram-negative bacterial peptidoglycan. Science.

[B117] Girardin SE, Boneca IG, Viala J, Chamaillard M, Labigne A, Thomas G, Philpott DJ, Sansonetti PJ (2003). Nod2 is a general sensor of peptidoglycan through muramyl dipeptide (MDP) detection. J Biol Chem.

[B118] Inohara N, Ogura Y, Fontalba A, Gutierrez O, Pons F, Crespo J, Fukase K, Inamura S, Kusumoto S, Hashimoto M, Foster SJ, Moran AP, Fernandez-Luna JL, Nunez G (2003). Host recognition of bacterial muramyl dipeptide mediated through NOD2. Implications for Crohn's disease. J Biol Chem.

[B119] Cossart P, Sansonetti PJ (2004). Bacterial invasion: the paradigms of enteroinvasive pathogens. Science.

[B120] Vavricka SR, Musch MW, Chang JE, Nakagawa Y, Phanvijhitsiri K, Waypa TS, Merlin D, Schneewind O, Chang EB (2004). hPepT1 transports muramyl dipeptide, activating NF-kappaB and stimulating IL-8 secretion in human colonic Caco2/bbe cells. Gastroenterology.

[B121] Groneberg DA, Fischer A, Chung KF, Daniel H (2004). Molecular mechanisms of pulmonary peptidomimetic drug and peptide transport. Am J Respir Cell Mol Biol.

[B122] Barnich N, Aguirre JE, Reinecker HC, Xavier R, Podolsky DK (2005). Membrane recruitment of NOD2 in intestinal epithelial cells is essential for nuclear factor-{kappa}B activation in muramyl dipeptide recognition. J Cell Biol.

[B123] Travassos LH, Carneiro LA, Girardin SE, Boneca IG, Lemos R, Bozza MT, Domingues RC, Coyle AJ, Bertin J, Philpott DJ, Plotkowski MC (2005). Nod1 participates in the innate immune response to Pseudomonas aeruginosa. J Biol Chem.

[B124] Ferwerda G, Girardin SE, Kullberg BJ, Le Bourhis L, de Jong DJ, Langenberg DM, van Crevel R, Adema GJ, Ottenhoff TH, Van der Meer JW, Netea MG (2005). NOD2 and toll-like receptors are nonredundant recognition systems of Mycobacterium tuberculosis. PLoS Pathog.

[B125] Opitz B, Forster S, Hocke AC, Maass M, Schmeck B, Hippenstiel S, Suttorp N, Krull M (2005). Nod1-mediated endothelial cell activation by Chlamydophila pneumoniae. Circ Res.

[B126] Opitz B, Puschel A, Beermann W, Hocke AC, Forster S, Schmeck B, van LV, Chakraborty T, Suttorp N, Hippenstiel S (2006). Listeria monocytogenes Activated p38 MAPK and Induced IL-8 Secretion in a Nucleotide-Binding Oligomerization Domain 1-Dependent Manner in Endothelial Cells. J Immunol.

[B127] Hisamatsu T, Suzuki M, Podolsky DK (2003). Interferon-gamma augments CARD4/NOD1 gene and protein expression through interferon regulatory factor-1 in intestinal epithelial cells. J Biol Chem.

[B128] Rosenstiel P, Fantini M, Brautigam K, Kuhbacher T, Waetzig GH, Seegert D, Schreiber S (2003). TNF-alpha and IFN-gamma regulate the expression of the NOD2 (CARD15) gene in human intestinal epithelial cells. Gastroenterology.

[B129] Diez E, Lee SH, Gauthier S, Yaraghi Z, Tremblay M, Vidal S, Gros P (2003). Birc1e is the gene within the Lgn1 locus associated with resistance to Legionella pneumophila. Nat Genet.

[B130] Wright EK, Goodart SA, Growney JD, Hadinoto V, Endrizzi MG, Long EM, Sadigh K, Abney AL, Bernstein-Hanley I, Dietrich WF (2003). Naip5 affects host susceptibility to the intracellular pathogen Legionella pneumophila. Curr Biol.

[B131] Molofsky AB, Byrne BG, Whitfield NN, Madigan CA, Fuse ET, Tateda K, Swanson MS (2006). Cytosolic recognition of flagellin by mouse macrophages restricts Legionella pneumophila infection. J Exp Med.

[B132] Ren T, Zamboni DS, Roy CR, Dietrich WF, Vance RE (2006). Flagellin-Deficient Legionella Mutants Evade Caspase-1- and Naip5-Mediated Macrophage Immunity. PLoS Pathog.

[B133] Zamboni DS, Kobayashi KS, Kohlsdorf T, Ogura Y, Long EM, Vance RE, Kuida K, Mariathasan S, Dixit VM, Flavell RA, Dietrich WF, Roy CR (2006). The Birc1e cytosolic pattern-recognition receptor contributes to the detection and control of Legionella pneumophila infection. Nat Immunol.

[B134] Chamaillard M, Girardin SE, Viala J, Philpott DJ (2003). Nods, Nalps and Naip: intracellular regulators of bacterial-induced inflammation. Cell Microbiol.

[B135] Ting JP, Davis BK (2005). CATERPILLER: a novel gene family important in immunity, cell death, and diseases. Annu Rev Immunol.

[B136] Kanneganti TD, Ozoren N, Body-Malapel M, Amer A, Park JH, Franchi L, Whitfield J, Barchet W, Colonna M, Vandenabeele P, Bertin J, Coyle A, Grant EP, Akira S, Nunez G (2006). Bacterial RNA and small antiviral compounds activate caspase-1 through cryopyrin/Nalp3. Nature.

[B137] Mariathasan S, Weiss DS, Newton K, McBride J, O'rourke K, Roose-Girma M, Lee WP, Weinrauch Y, Monack DM, Dixit VM (2006). Cryopyrin activates the inflammasome in response to toxins and ATP. Nature.

[B138] Martinon F, Agostini L, Meylan E, Tschopp J (2004). Identification of bacterial muramyl dipeptide as activator of the NALP3/cryopyrin inflammasome. Curr Biol.

[B139] Sutterwala FS, Ogura Y, Szczepanik M, Lara-Tejero M, Lichtenberger GS, Grant EP, Bertin J, Coyle AJ, Galan JE, Askenase PW, Flavell RA (2006). Critical role for NALP3/CIAS1/Cryopyrin in innate and adaptive immunity through its regulation of caspase-1. Immunity.

[B140] Netea MG, Ferwerda G, de Jong DJ, Jansen T, Jacobs L, Kramer M, Naber TH, Drenth JP, Girardin SE, Kullberg BJ, Adema GJ, Van der Meer JW (2005). Nucleotide-binding oligomerization domain-2 modulates specific TLR pathways for the induction of cytokine release. J Immunol.

[B141] Tada H, Aiba S, Shibata K, Ohteki T, Takada H (2005). Synergistic effect of Nod1 and Nod2 agonists with toll-like receptor agonists on human dendritic cells to generate interleukin-12 and T helper type 1 cells. Infect Immun.

[B142] Traub S, Kubasch N, Morath S, Kresse M, Hartung T, Schmidt RR, Hermann C (2004). Structural requirements of synthetic muropeptides to synergize with lipopolysaccharide in cytokine induction. J Biol Chem.

[B143] Uehara A, Yang S, Fujimoto Y, Fukase K, Kusumoto S, Shibata K, Sugawara S, Takada H (2005). Muramyldipeptide and diaminopimelic acid-containing desmuramylpeptides in combination with chemically synthesized Toll-like receptor agonists synergistically induced production of interleukin-8 in a NOD2- and NOD1-dependent manner, respectively, in human monocytic cells in culture. Cell Microbiol.

[B144] Williams KL, Lich JD, Duncan JA, Reed W, Rallabhandi P, Moore C, Kurtz S, Coffield VM, Accavitti-Loper MA, Su L, Vogel SN, Braunstein M, Ting JP (2005). The CATERPILLER protein monarch-1 is an antagonist of toll-like receptor-, tumor necrosis factor alpha-, and Mycobacterium tuberculosis-induced pro-inflammatory signals. J Biol Chem.

[B145] Kobayashi KS, Chamaillard M, Ogura Y, Henegariu O, Inohara N, Nunez G, Flavell RA (2005). Nod2-dependent regulation of innate and adaptive immunity in the intestinal tract. Science.

[B146] Maeda S, Hsu LC, Liu H, Bankston LA, Iimura M, Kagnoff MF, Eckmann L, Karin M (2005). Nod2 mutation in Crohn's disease potentiates NF-kappaB activity and IL-1beta processing. Science.

[B147] Watanabe T, Kitani A, Murray PJ, Strober W (2004). NOD2 is a negative regulator of Toll-like receptor 2-mediated T helper type 1 responses. Nat Immunol.

[B148] Matikainen S, Siren J, Tissari J, Veckman V, Pirhonen J, Severa M, Sun Q, Lin R, Meri S, Uze G, Hiscott J, Julkunen I (2006). Tumor necrosis factor alpha enhances influenza A virus-induced expression of antiviral cytokines by activating RIG-I gene expression. J Virol.

[B149] Kato H, Takeuchi O, Sato S, Yoneyama M, Yamamoto M, Matsui K, Uematsu S, Jung A, Kawai T, Ishii KJ, Yamaguchi O, Otsu K, Tsujimura T, Koh CS, Sousa R, Matsuura Y, Fujita T, Akira S (2006). Differential roles of MDA5 and RIG-I helicases in the recognition of RNA viruses. Nature.

[B150] Barnes B, Lubyova B, Pitha PM (2002). On the role of IRF in host defense. J Interferon Cytokine Res.

[B151] Honda K, Yanai H, Takaoka A, Taniguchi T (2005). Regulation of the type I IFN induction: a current view. Int Immunol.

[B152] Perry AK, Chen G, Zheng D, Tang H, Cheng G (2005). The host type I interferon response to viral and bacterial infections. Cell Res.

[B153] Kawai T, Adachi O, Ogawa T, Takeda K, Akira S (1999). Unresponsiveness of MyD88-deficient mice to endotoxin. Immunity.

[B154] Medzhitov R, Preston-Hurlburt P, Kopp E, Stadlen A, Chen C, Ghosh S, Janeway CAJ (1998). MyD88 is an adaptor protein in the hToll/IL-1 receptor family signaling pathways. Mol Cell.

[B155] Fitzgerald KA, Rowe DC, Barnes BJ, Caffrey DR, Visintin A, Latz E, Monks B, Pitha PM, Golenbock DT (2003). LPS-TLR4 signaling to IRF-3/7 and NF-kappaB involves the toll adapters TRAM and TRIF. J Exp Med.

[B156] Hoebe K, Du X, Georgel P, Janssen E, Tabeta K, Kim SO, Goode J, Lin P, Mann N, Mudd S, Crozat K, Sovath S, Han J, Beutler B (2003). Identification of Lps2 as a key transducer of MyD88-independent TIR signalling. Nature.

[B157] Yamamoto M, Sato S, Hemmi H, Hoshino K, Kaisho T, Sanjo H, Takeuchi O, Sugiyama M, Okabe M, Takeda K, Akira S (2003). Role of adaptor TRIF in the MyD88-independent toll-like receptor signaling pathway. Science.

[B158] Yamamoto M, Sato S, Hemmi H, Uematsu S, Hoshino K, Kaisho T, Takeuchi O, Takeda K, Akira S (2003). TRAM is specifically involved in the Toll-like receptor 4-mediated MyD88-independent signaling pathway. Nat Immunol.

[B159] Fitzgerald KA, Palsson-McDermott EM, Bowie AG, Jefferies CA, Mansell AS, Brady G, Brint E, Dunne A, Gray P, Harte MT, McMurray D, Smith DE, Sims JE, Bird TA, O'Neill LA (2001). Mal (MyD88-adapter-like) is required for Toll-like receptor-4 signal transduction. Nature.

[B160] Horng T, Barton GM, Flavell RA, Medzhitov R (2002). The adaptor molecule TIRAP provides signalling specificity for Toll-like receptors. Nature.

[B161] Honda K, Ohba Y, Yanai H, Negishi H, Mizutani T, Takaoka A, Taya C, Taniguchi T (2005). Spatiotemporal regulation of MyD88-IRF-7 signalling for robust type-I interferon induction. Nature.

[B162] Kawai T, Sato S, Ishii KJ, Coban C, Hemmi H, Yamamoto M, Terai K, Matsuda M, Inoue J, Uematsu S, Takeuchi O, Akira S (2004). Interferon-alpha induction through Toll-like receptors involves a direct interaction of IRF7 with MyD88 and TRAF6. Nat Immunol.

[B163] Schoenemeyer A, Barnes BJ, Mancl ME, Latz E, Goutagny N, Pitha PM, Fitzgerald KA, Golenbock DT (2005). The interferon regulatory factor, IRF5, is a central mediator of toll-like receptor 7 signaling. J Biol Chem.

[B164] Takaoka A, Yanai H, Kondo S, Duncan G, Negishi H, Mizutani T, Kano S, Honda K, Ohba Y, Mak TW, Taniguchi T (2005). Integral role of IRF-5 in the gene induction programme activated by Toll-like receptors. Nature.

[B165] Uematsu S, Sato S, Yamamoto M, Hirotani T, Kato H, Takeshita F, Matsuda M, Coban C, Ishii KJ, Kawai T, Takeuchi O, Akira S (2005). Interleukin-1 receptor-associated kinase-1 plays an essential role for Toll-like receptor (TLR)7- and TLR9-mediated interferon-{alpha} induction. J Exp Med.

[B166] Bonizzi G, Karin M (2004). The two NF-kappaB activation pathways and their role in innate and adaptive immunity. Trends Immunol.

[B167] Schmeck B, Zahlten J, Moog K, van LV, Huber S, Hocke AC, Opitz B, Hoffmann E, Kracht M, Zerrahn J, Hammerschmidt S, Rosseau S, Suttorp N, Hippenstiel S (2004). Streptococcus pneumoniae-induced p38 MAPK-dependent phosphorylation of RelA at the interleukin-8 promotor. J Biol Chem.

[B168] Slevogt H, Schmeck B, Jonatat C, Zahlten J, Beermann W, van LV, Opitz B, Dietel S, Dje NP, Hippenstiel S, Suttorp N, Seybold J (2006). MORAXELLA CATARRHALIS INDUCES INFLAMMATORY RESPONSE OF BRONCHIAL EPITHELIAL CELLS VIA MITOGEN-ACTIVATED PROTEIN KINASE AND NF-{kappa}B ACTIVATION AND HISTONE DEACETYLASE ACTIVITY REDUCTION. Am J Physiol Lung Cell Mol Physiol.

[B169] Wang J, Gigliotti F, Maggirwar S, Johnston C, Finkelstein JN, Wright TW (2005). Pneumocystis carinii activates the NF-kappaB signaling pathway in alveolar epithelial cells. Infect Immun.

[B170] Joseph T, Look D, Ferkol T (2005). NF-kappaB activation and sustained IL-8 gene expression in primary cultures of cystic fibrosis airway epithelial cells stimulated with Pseudomonas aeruginosa. Am J Physiol Lung Cell Mol Physiol.

[B171] Poynter ME, Irvin CG, Janssen-Heininger YM (2003). A prominent role for airway epithelial NF-kappa B activation in lipopolysaccharide-induced airway inflammation. J Immunol.

[B172] Chin AI, Dempsey PW, Bruhn K, Miller JF, Xu Y, Cheng G (2002). Involvement of receptor-interacting protein 2 in innate and adaptive immune responses. Nature.

[B173] Kobayashi K, Inohara N, Hernandez LD, Galan JE, Nunez G, Janeway CA, Medzhitov R, Flavell RA (2002). RICK/Rip2/CARDIAK mediates signalling for receptors of the innate and adaptive immune systems. Nature.

[B174] Sadikot RT, Han W, Everhart MB, Zoia O, Peebles RS, Jansen ED, Yull FE, Christman JW, Blackwell TS (2003). Selective I kappa B kinase expression in airway epithelium generates neutrophilic lung inflammation. J Immunol.

[B175] Sadikot RT, Zeng H, Joo M, Everhart MB, Sherrill TP, Li B, Cheng DS, Yull FE, Christman JW, Blackwell TS (2006). Targeted immunomodulation of the NF-kappaB pathway in airway epithelium impacts host defense against Pseudomonas aeruginosa. J Immunol.

[B176] Fitzgerald KA, McWhirter SM, Faia KL, Rowe DC, Latz E, Golenbock DT, Coyle AJ, Liao SM, Maniatis T (2003). IKKepsilon and TBK1 are essential components of the IRF3 signaling pathway. Nat Immunol.

[B177] Kawai T, Takahashi K, Sato S, Coban C, Kumar H, Kato H, Ishii KJ, Takeuchi O, Akira S (2005). IPS-1, an adaptor triggering RIG-I- and Mda5-mediated type I interferon induction. Nat Immunol.

[B178] Meylan E, Curran J, Hofmann K, Moradpour D, Binder M, Bartenschlager R, Tschopp J (2005). Cardif is an adaptor protein in the RIG-I antiviral pathway and is targeted by hepatitis C virus. Nature.

[B179] Sarkar SN, Peters KL, Elco CP, Sakamoto S, Pal S, Sen GC (2004). Novel roles of TLR3 tyrosine phosphorylation and PI3 kinase in double-stranded RNA signaling. Nat Struct Mol Biol.

[B180] Seth RB, Sun L, Ea CK, Chen ZJ (2005). Identification and characterization of MAVS, a mitochondrial antiviral signaling protein that activates NF-kappaB and IRF 3. Cell.

[B181] Sharma S, tenOever BR, Grandvaux N, Zhou GP, Lin R, Hiscott J (2003). Triggering the interferon antiviral response through an IKK-related pathway. Science.

[B182] Xu LG, Wang YY, Han KJ, Li LY, Zhai Z, Shu HB (2005). VISA is an adapter protein required for virus-triggered IFN-beta signaling. Mol Cell.

[B183] Decker T, Muller M, Stockinger S (2005). The yin and yang of type I interferon activity in bacterial infection. Nat Rev Immunol.

[B184] Theofilopoulos AN, Baccala R, Beutler B, Kono DH (2005). Type I interferons (alpha/beta) in immunity and autoimmunity. Annu Rev Immunol.

[B185] Dunne A, O'Neill LA (2003). The interleukin-1 receptor/Toll-like receptor superfamily: signal transduction during inflammation and host defense. Sci STKE.

[B186] Girardin SE, Tournebize R, Mavris M, Page AL, Li X, Stark GR, Bertin J, DiStefano PS, Yaniv M, Sansonetti PJ, Philpott DJ (2001). CARD4/Nod1 mediates NF-kappaB and JNK activation by invasive Shigella flexneri. EMBO Rep.

[B187] Alcorn MJ, Booth JL, Coggeshall KM, Metcalf JP (2001). Adenovirus type 7 induces interleukin-8 production via activation of extracellular regulated kinase 1/2. J Virol.

[B188] Avni O, Lee D, Macian F, Szabo SJ, Glimcher LH, Rao A (2002). T(H) cell differentiation is accompanied by dynamic changes in histone acetylation of cytokine genes. Nat Immunol.

[B189] Muegge K (2002). Preparing the target for the bullet. Nat Immunol.

[B190] Strahl BD, Allis CD (2000). The language of covalent histone modifications. Nature.

[B191] Claus R, Lubbert M (2003). Epigenetic targets in hematopoietic malignancies. Oncogene.

[B192] Jenuwein T, Allis CD (2001). Translating the histone code. Science.

[B193] Saccani S, Pantano S, Natoli G (2002). p38-Dependent marking of inflammatory genes for increased NF-kappa B recruitment. Nat Immunol.

[B194] Schmeck B, Beermann W, van LV, Zahlten J, Opitz B, Witzenrath M, Hocke AC, Chakraborty T, Kracht M, Rosseau S, Suttorp N, Hippenstiel S (2005). Intracellular bacteria differentially regulated endothelial cytokine release by MAPK-dependent histone modification. J Immunol.

[B195] Barnes PJ, Adcock IM, Ito K (2005). Histone acetylation and deacetylation: importance in inflammatory lung diseases. Eur Respir J.

[B196] Ito K, Ito M, Elliott WM, Cosio B, Caramori G, Kon OM, Barczyk A, Hayashi S, Adcock IM, Hogg JC, Barnes PJ (2005). Decreased histone deacetylase activity in chronic obstructive pulmonary disease. N Engl J Med.

[B197] Ulanova M, Puttagunta L, Marcet-Palacios M, Duszyk M, Steinhoff U, Duta F, Kim MK, Indik ZK, Schreiber AD, Befus AD (2005). Syk tyrosine kinase participates in beta1-integrin signaling and inflammatory responses in airway epithelial cells. Am J Physiol Lung Cell Mol Physiol.

[B198] Hippenstiel S, Kratz T, Krull M, Seybold J, Eichel-Streiber C, Suttorp N (1998). Rho protein inhibition blocks protein kinase C translocation and activation. Biochem Biophys Res Commun.

[B199] Stark JM, Stark MA, Colasurdo GN, LeVine AM (2006). Decreased bacterial clearance from the lungs of mice following primary respiratory syncytial virus infection. J Med Virol.

[B200] LeVine AM, Koeningsknecht V, Stark JM (2001). Decreased pulmonary clearance of S. pneumoniae following influenza A infection in mice. J Virol Methods.

[B201] Ratner AJ, Lysenko ES, Paul MN, Weiser JN (2005). Synergistic proinflammatory responses induced by polymicrobial colonization of epithelial surfaces. Proc Natl Acad Sci U S A.

[B202] Groskreutz DJ, Monick MM, Powers LS, Yarovinsky TO, Look DC, Hunninghake GW (2006). Respiratory syncytial virus induces TLR3 protein and protein kinase R, leading to increased double-stranded RNA responsiveness in airway epithelial cells. J Immunol.

[B203] Bals R, Hiemstra PS (2004). Innate immunity in the lung: how epithelial cells fight against respiratory pathogens. Eur Respir J.

[B204] Strieter RM, Belperio JA, Keane MP (2003). Host innate defenses in the lung: the role of cytokines. Curr Opin Infect Dis.

[B205] Strieter RM (2002). Interleukin-8: a very important chemokine of the human airway epithelium. Am J Physiol Lung Cell Mol Physiol.

[B206] Cooper P, Potter S, Mueck B, Yousefi S, Jarai G (2001). Identification of genes induced by inflammatory cytokines in airway epithelium. Am J Physiol Lung Cell Mol Physiol.

[B207] Neff SB, Z'graggen BR, Neff TA, Jamnicki-Abegg M, Suter D, Schimmer RC, Booy C, Joch H, Pasch T, Ward PA, Beck-Schimmer B (2006). Inflammatory response of tracheobronchial epithelial cells to endotoxin. Am J Physiol Lung Cell Mol Physiol.

[B208] Pichavant M, Delneste Y, Jeannin P, Fourneau C, Brichet A, Tonnel AB, Gosset P (2003). Outer membrane protein A from Klebsiella pneumoniae activates bronchial epithelial cells: implication in neutrophil recruitment. J Immunol.

[B209] Yu ML, Limper AH (1997). Pneumocystis carinii induces ICAM-1 expression in lung epithelial cells through a TNF-alpha-mediated mechanism. Am J Physiol.

[B210] Chung KF, Barnes PJ (1999). Cytokines in asthma. Thorax.

[B211] De Smet K, Contreras R (2005). Human antimicrobial peptides: defensins, cathelicidins and histatins. Biotechnol Lett.

[B212] Koczulla AR, Bals R (2003). Antimicrobial peptides: current status and therapeutic potential. Drugs.

[B213] Travis SM, Singh PK, Welsh MJ (2001). Antimicrobial peptides and proteins in the innate defense of the airway surface. Curr Opin Immunol.

[B214] Harder J, Meyer-Hoffert U, Teran LM, Schwichtenberg L, Bartels J, Maune S, Schroder JM (2000). Mucoid Pseudomonas aeruginosa, TNF-alpha, and IL-1beta, but not IL-6, induce human beta-defensin-2 in respiratory epithelia. Am J Respir Cell Mol Biol.

[B215] N'Guessan PD, Hippenstiel S, Etouem MO, Zahlten J, Beermann W, Lindner D, Opitz B, Witzenrath M, Rosseau S, Suttorp N, Schmeck B (2006). Streptococcus pneumoniae-induced p38 MAPK- and NF-{kappa}B-dependent COX-2 expression in human lung epithelium. Am J Physiol Lung Cell Mol Physiol.

[B216] Klockmann MT, Jahn HU, Hippenstiel S, Kramer HJ, Suttorp N (1998). Interaction of human neutrophils with airway epithelial cells: reduction of leukotriene B4 generation by epithelial cell derived prostaglandin E2. J Cell Physiol.

[B217] Lambrecht BN, Prins JB, Hoogsteden HC (2001). Lung dendritic cells and host immunity to infection. Eur Respir J.

[B218] Moore BB, Moore TA, Toews GB (2001). Role of T- and B-lymphocytes in pulmonary host defences. Eur Respir J.

[B219] Kadioglu A, Andrew PW (2004). The innate immune response to pneumococcal lung infection: the untold story. Trends Immunol.

[B220] Delclaux C, Azoulay E (2003). Inflammatory response to infectious pulmonary injury. Eur Respir J Suppl.

[B221] Nicod LP (1999). Pulmonary defence mechanisms. Respiration.

[B222] Hajjar AM, Harowicz H, Liggitt HD, Fink PJ, Wilson CB, Skerrett SJ (2005). An essential role for non-bone marrow-derived cells in control of Pseudomonas aeruginosa pneumonia. Am J Respir Cell Mol Biol.

[B223] Noulin N, Quesniaux VF, Schnyder-Candrian S, Schnyder B, Maillet I, Robert T, Vargaftig BB, Ryffel B, Couillin I (2005). Both hemopoietic and resident cells are required for MyD88-dependent pulmonary inflammatory response to inhaled endotoxin. J Immunol.

[B224] Hollingsworth JW, Chen BJ, Brass DM, Berman K, Gunn MD, Cook DN, Schwartz DA (2005). The Critical Role of Hematopoietic Cells in Lipopolysaccharide-induced Airway Inflammation. Am J Respir Crit Care Med.

[B225] Skerrett SJ, Liggitt HD, Hajjar AM, Ernst RK, Miller SI, Wilson CB (2004). Respiratory epithelial cells regulate lung inflammation in response to inhaled endotoxin. Am J Physiol Lung Cell Mol Physiol.

[B226] Hu M, Miller EJ, Lin X, Simms HH (2004). Transmigration across a lung epithelial monolayer delays apoptosis of polymorphonuclear leukocytes. Surgery.

[B227] Hu M, Lin X, Du Q, Miller EJ, Wang P, Simms HH (2005). Regulation of polymorphonuclear leukocyte apoptosis: role of lung endothelium-epithelium bilayer transmigration. Am J Physiol Lung Cell Mol Physiol.

[B228] Paine RIII, Morris SB, Jin H, Baleeiro CE, Wilcoxen SE (2002). ICAM-1 facilitates alveolar macrophage phagocytic activity through effects on migration over the AEC surface. Am J Physiol Lung Cell Mol Physiol.

[B229] Tsutsumi-Ishii Y, Nagaoka I (2003). Modulation of human beta-defensin-2 transcription in pulmonary epithelial cells by lipopolysaccharide-stimulated mononuclear phagocytes via proinflammatory cytokine production. J Immunol.

[B230] Aarbiou J, Ertmann M, van Wetering S, van Noort P, Rook D, Rabe KF, Litvinov SV, van Krieken JH, de Boer WI, Hiemstra PS (2002). Human neutrophil defensins induce lung epithelial cell proliferation in vitro. J Leukoc Biol.

[B231] van Wetering S, Mannesse-Lazeroms SP, van Sterkenburg MA, Hiemstra PS (2002). Neutrophil defensins stimulate the release of cytokines by airway epithelial cells: modulation by dexamethasone. Inflamm Res.

[B232] van Wetering S, Tjabringa GS, Hiemstra PS (2005). Interactions between neutrophil-derived antimicrobial peptides and airway epithelial cells. J Leukoc Biol.

[B233] Liew FY, Xu D, Brint EK, O'Neill LA (2005). Negative regulation of toll-like receptor-mediated immune responses. Nat Rev Immunol.

[B234] Chaby R, Garcia-Verdugo I, Espinassous Q, Augusto LA (2005). Interactions between LPS and lung surfactant proteins. J Endotoxin Res.

[B235] Meyer KC, Zimmerman JJ (2002). Inflammation and surfactant. Paediatr Respir Rev.

[B236] Sano H, Kuroki Y (2005). The lung collectins, SP-A and SP-D, modulate pulmonary innate immunity. Mol Immunol.

[B237] Yoshida M, Whitsett JA (2004). Interactions between pulmonary surfactant and alveolar macrophages in the pathogenesis of lung disease. Cell Mol Biol (Noisy -le-grand).

[B238] Dillon CP, Sandy P, Nencioni A, Kissler S, Rubinson DA, Van Parijs L (2004). RNAi as an Experimental and Therapeutic Tool to Study and Regulate Physiological and Disease Processes. Annu Rev Physiol.

[B239] Prawitt D, Brixel L, Spangenberg C, Eshkind L, Heck R, Oesch F, Zabel B, Bockamp E (2004). RNAi knock-down mice: an emerging technology for post-genomic functional genetics. Cytogenet Genome Res.

[B240] Bitko V, Musiyenko A, Shulyayeva O, Barik S (2005). Inhibition of respiratory viruses by nasally administered siRNA. Nat Med.

[B241] Griffiths MJ, Bonnet D, Janes SM (2005). Stem cells of the alveolar epithelium. Lancet.

[B242] Tesfaigzi Y (2003). Processes involved in the repair of injured airway epithelia. Arch Immunol Ther Exp (Warsz ).

[B243] Shimabukuro DW, Sawa T, Gropper MA (2003). Injury and repair in lung and airways. Crit Care Med.

[B244] Abreu MT, Fukata M, Arditi M (2005). TLR signaling in the gut in health and disease. J Immunol.

[B245] Cario E, Podolsky DK (2005). Intestinal epithelial TOLLerance versus inTOLLerance of commensals. Mol Immunol.

[B246] Mueller-Anneling L, Avol E, Peters JM, Thorne PS (2004). Ambient endotoxin concentrations in PM10 from Southern California. Environ Health Perspect.

[B247] Spaan S, Wouters IM, Oosting I, Doekes G, Heederik D (2006). Exposure to inhalable dust and endotoxins in agricultural industries. J Environ Monit.

[B248] Lane SR, Nicholls PJ, Sewell RD (2004). The measurement and health impact of endotoxin contamination in organic dusts from multiple sources: focus on the cotton industry. Inhal Toxicol.

[B249] Jia HP, Kline JN, Penisten A, Apicella MA, Gioannini TL, Weiss J, McCray PBJ (2004). Endotoxin responsiveness of human airway epithelia is limited by low expression of MD-2. Am J Physiol Lung Cell Mol Physiol.

[B250] Zhang G, Ghosh S (2002). Negative regulation of toll-like receptor-mediated signaling by Tollip. J Biol Chem.

[B251] Burns K, Clatworthy J, Martin L, Martinon F, Plumpton C, Maschera B, Lewis A, Ray K, Tschopp J, Volpe F (2000). Tollip, a new component of the IL-1RI pathway, links IRAK to the IL-1 receptor. Nat Cell Biol.

[B252] Apisarnthanarak A, Mundy LM (2005). Etiology of community-acquired pneumonia. Clin Chest Med.

[B253] Sinaniotis CA (2004). Viral pneumoniae in children: incidence and aetiology. Paediatr Respir Rev.

